# TGM2-mediated serotonylation of GPX4 confers ferroptosis resistance to promote gastric tumorigenesis

**DOI:** 10.1038/s41421-026-00885-6

**Published:** 2026-04-28

**Authors:** Junping Bai, Dandan Geng, Xinwen Chen, Wanting Li, Xiaowei Wang, Luyang Tian, Yi Han, Zhao Jin, Meihang Du, Yang Tang, Weisheng Hu, Chunxiao Zhu, Shan Zhang, Zhangting Zhao, Run Zhang, Xinru Zhang, Wei Kang, KaFai To, Sachiyo Nomura, Fenghua Guo, Shi Jiao, Yixuan Xie, Zhaocai Zhou, Chao Dong, Hui Li, Liwei An

**Affiliations:** 1 Department of Stomatology, Shanghai Tenth People’s Hospital, Department of Biochemistry and Molecular Biology, Tongji University Cancer Center, School of Medicine, Tongji University, Shanghai, China; 2 School of Medicine, Anhui University of Science and Technology, Huainan, Anhui China; 3 Department of Gastrointestinal Cancer Biology, Tianjin Medical University Cancer Institute and Hospital, National Clinical Research Center for Cancer, Tianjin Key Laboratory of Digestive Cancer, Tianjin, China; 4 School of Basic Medicine and Clinical Pharmacy, China Pharmaceutical University, Nanjing, Jiangsu China; 5 State Key Laboratory of Genetics and Development of Complex Phenotypes, School of Life Sciences, Zhongshan Hospital, Fudan University, Shanghai, China; 6 Institutes of Biomedical Sciences, Inner Mongolia University, Hohhot, Inner Mongolia Autonomous Region China; 7 NHC Key Laboratory of Glycoconjugates Research, Department of Biochemistry and Molecular Biology, School of Basic Medical Sciences, Fudan University, Shanghai, China; 8 Shanghai Bioprofile Technolgy Co. Ltd, Shanghai, China; 9 Department of Anatomical and Cellular Pathology, State Key Laboratory of Translational Oncology, State Key Laboratory of Digestive Disease, Prince of Wales Hospital, The Chinese University of Hong Kong, Hong Kong SAR, China; 10 Department of Gastrointestinal surgery, Graduate School of Medicine, The University of Tokyo, Tokyo, Japan; 11 Department of General Surgery, Huashan Hospital, Fudan University Shanghai Medical College, Shanghai, China; 12 Collaborative Innovation Center for Cancer Personalized Medicine, School of Public Health, Nanjing Medical University, Nanjing, Jiangsu China; 13 Tianfu Jincheng Laboratory, Chengdu, Sichuan China; 14 Department of Medical Oncology, The First Affiliated Hospital of Kunming Medical University, Kunming, Yunnan China

**Keywords:** Post-translational modifications, Gastric cancer

## Abstract

Protein monoaminylation represents a new layer of neural–cancer regulation, but its role in gastric tumorigenesis is not understood. Using untargeted plasma metabolomics, we revealed that the level of serotonin (5-HT) is significantly elevated in gastric cancer (GC) patients. Functionally, 5-HT treatment dramatically promoted GC cell proliferation and tumor growth in a dose-dependent manner. Importantly, this oncogenic effect was abrogated by the inhibition of transglutaminase 2 (TGM2), indicating a crucial role for protein serotonylation via a receptor-independent mechanism. Using a 5-HT-based chemoproteomic probe, we identified a broad spectrum of serotonylation targets, including key ferroptosis-related proteins such as glutathione peroxidase 4 (GPX4). Specifically, we found that GPX4 is serotonylated by TGM2 at residues Gln55 and Gln77, which increases GPX4 protein stability by attenuating its ubiquitin-mediated degradation, thereby conferring resistance to ferroptosis and facilitating tumor growth. Clinically, TGM2 levels were positively correlated with tumoral GPX4 expression in GC patient specimens. Collectively, our results establish TGM2-mediated GPX4 serotonylation as a key mechanism driving GC progression through ferroptosis resistance, highlighting its potential as both a diagnostic biomarker and a therapeutic target within the neural–tumor axis.

## Introduction

Gastric cancer (GC) is as the fifth most common malignancy and a leading cause of cancer-related mortality worldwide^1^. The development and progression of GC involve complex, multifactorial processes, posing significant challenges for the design of next-generation targeted therapies^2^. Accumulating evidence highlights the pivotal role of the nervous system in carcinogenesis^3^, with neural innervation now recognized as a hallmark of cancer^4^. In particular, local neuronal infiltration is frequently observed in tumor tissues and is positively correlated with disease progression and poor patient prognosis^5–7^. However, the precise mechanisms governing nerve–cancer crosstalk, especially the contribution of neurotransmitters to GC pathogenesis, remain incompletely understood.

Serotonin (5-hydroxytryptamine, 5-HT) is a monoamine neurotransmitter synthesized from tryptophan via sequential reactions catalyzed by tryptophan hydroxylase (TPH) and DOPA decarboxylase (DDC)^8^. In mammals, peripheral and central 5-HT pools are spatially segregated by the blood–brain barrier, with its synthesis primarily mediated by TPH1 and TPH2, respectively^9,10^. Notably, peripheral 5-HT is largely produced by enterochromaffin cells through TPH1 catalysis and constitutes ~95% of the body’s total 5-HT, highlighting its crucial role in both physiological and pathological processes in peripheral tissues^11^. In addition to its classical role in regulating gastrointestinal motility^12^, emerging evidence indicates that 5-HT also promotes tumor proliferation, invasion, metastasis, and angiogenesis^13^.

The oncogenic effects of serotonin have traditionally been attributed to the activation of specific 5-HT receptors (5-HTRs) on target cells^14^, and serotonin has been implicated in the progression of several cancers, including those of the breast, colorectum, and liver^15^. Notably, HTR2B overexpression in GC patients has been linked to clinical prognosis^16^. In addition to affecting canonical receptor signaling, 5-HT also exerts receptor-independent effects via transglutaminase 2 (TGM2)-catalyzed serotonylation, a covalent post-translational modification that modulates substrate protein function^17,18^. For instance, serotonin promotes tumor progression by serotonylating histones (e.g., H3Q5ser) in cancer and immune cells, facilitating cancer growth, metastasis, and immune responses through metabolic reprogramming^19–22^. Similarly, the serotonylation of non-histone proteins such as GAPDH links glycolytic flux to antitumor immunity in CD8⁺ T cells^23^. Thus, peripheral 5-HT may influence tumorigenesis through both receptor-dependent and receptor-independent mechanisms, underscoring the multifaceted role of neurotransmitter signaling within the tumor microenvironment.

Untargeted metabolomic profiling of plasma samples from a cohort of 41 GC patients revealed a significant increase in circulating 5-HT levels in GC patients compared with that in healthy controls. Functionally, 5-HT promoted cancer cell proliferation and tumor growth in a TGM2-dependent manner, an effect completely abolished by co-treatment with a TGM2 inhibitor, indicating a serotonylation-dependent mechanism. Using a 5-HT-based chemical probe 5-propargyltryptamide (5-PT) combined with quantitative proteomics, we comprehensively mapped the serotonylome in GC cells and identified glutathione peroxidase 4 (GPX4) as a novel substrate. Crucially, GPX4 serotonylation increases its protein stability, confers resistance to ferroptosis, and drives GC cell proliferation. Overall, TGM2 expression was found to be positively correlated with GPX4 levels in tumor specimens from GC patients, and our study reveals that serotonylation is a novel post-translational modification that regulates GPX4 and elucidates its functional significance in gastric tumorigenesis, providing mechanistic insights into neural–tumor interactions and revealing potential therapeutic targets.

## Results

### 5-HT promotes gastric tumor growth via a serotonylation-dependent mechanism

**Fig. 1:**
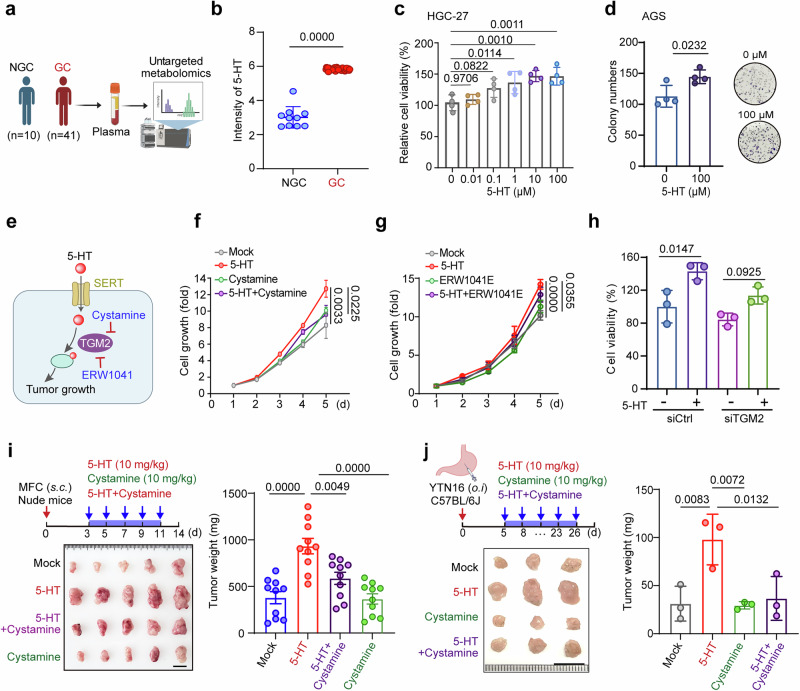
5-HT promotes gastric tumor growth via a serotonylation-dependent mechanism. **a** Workflow for untargeted mass spectrometry analysis of plasma from individuals without GC (NGC, *n* = 10) and GC patients (GC, *n* = 41). **b** Relative levels of 5-HT in plasma between normal controls (NGC, *n* = 10) and GC patients (GC, *n* = 41). **c**, **d** 5-HT promotes the proliferation of HGC-27 and AGS cells in vitro. AGS cells were first subjected to serum starvation for 6 h before they were treated with various concentrations of 5-HT for 48 h to assess relative cell viability (*n* = 4 per group; **c**). A colony formation assay of AGS cells was performed after treatment with various concentrations of 5-HT for 8 days (*n* = 4 per group, **d**). **e** The 5-HT signaling model shows that 5-HT promotes tumor progression via serotonylation pathways. **f** The relative viability of HGC-27 cells was measured after treatment with 5-HT (10 μM) or 5-HT combined with cystamine (100 μM) for various durations (*n* = 3 per group). **g** The relative viability of HGC-27 cells was measured after treatment with 5-HT (10 μM) or 5-HT combined with ERW1041E (50 μM) for various durations (*n* = 3 per group). **h** HGC-27 cells were transfected with siCtrl or siTGM2 for 24 h. After 6 h of serum starvation, these cells were treated with various concentrations of 5-HT for 24 h to assess their relative cell viability (*n* = 3 per group). **i** Schematic diagram of the workflow for analyzing subcutaneous MFC tumors (5 × 10^6^ cells) in nude mice treated with or without 5-HT (10 mg/kg) or 5-HT (10 mg/kg) combined with cystamine (10 mg/kg) for 10 days. Insert: Representative images of subcutaneous tumors are shown on the bottom. The weights of the subcutaneous MFC tumors in the nude mice are shown on the right (*n* = 10 per group). Scale bar, 1 cm. **j** Schematic diagram of the workflow. C57BL/6J mice were orthotopically injected with YTN16 cells (1 × 10^7^) treated with or without 5-HT (10 mg/kg) or 5-HT (10 mg/kg) combined with cysteamine (10 mg/kg). Insert: Representative images of orthotopic tumors are shown on the bottom. The weights of orthotopic gastric tumors in mice are shown on the right (*n* = 3 per group). Data are presented as mean ± SD from two or three independent experiments. Scale bar, 1 cm. Statistical analysis was performed using an unpaired *t* test (**b**, **d**), ordinary one-way ANOVA (**c**, **f**, **h**, **j**) or two-way ANOVA (**g**).

In an effort to comprehensively map metabolites associated with GC development, we performed untargeted metabolomic profiling of plasma samples from a cohort comprising 41 GC patients and 10 healthy controls (Fig. 1a). This approach revealed a significant increase in the plasma levels of several metabolites, particularly those involved in tryptophan metabolism (Supplementary Table S1). Notably, we observed a significant increase in the level of circulating 5-HT in GC patients compared with that in healthy controls (Fig. 1b; Supplementary Fig. S1a), which is consistent with the findings of a recent large-scale targeted metabolomics study involving 702 plasma samples from a multi-center GC cohort^24^.

We next asked whether such elevated 5-HT contributes to tumor progression. To this end, we treated the human GC cell line HGC-27 with increasing concentrations of 5-HT for 48 h and observed a dose-dependent increase in cell proliferation, as shown by both CCK-8 (Fig. 1c) and colony formation assays (Supplementary Fig. S1b, c). This pro-proliferative effect of 5-HT was also observed in another human GC line, AGS (Fig. 1d; Supplementary Fig. S1d), as well as in a mouse GC cell line, MFC (Supplementary Fig. S1e, f).

In addition to the well-characterized G protein-coupled receptor (e.g., HTR2B)-mediated signaling pathway in GC progression^16^, 5-HT can also regulate tumors through a post-translational modification known as protein serotonylation, which is primarily catalyzed by transglutaminase 2 (TGM2)^14,17^ (Fig. 1e). To determine whether TGM2-mediated serotonylation also contributes to the tumor-promoting effect of 5-HT, we pharmacologically inhibited TGM2 using two established inhibitors, cystamine^25^ and ERW1041E^26^. Both inhibitors markedly attenuated the pro-proliferative effect of 5-HT in HGC-27 cells (Fig. 1f, g). Moreover, we performed genetic validation by depleting TGM2 using a siRNA strategy (Supplementary Fig. S1g). Genetic knockdown of TGM2 via siRNA resulted in a similar attenuation of 5-HT-induced proliferation in HGC-27 cells (Fig. 1h).

Next, we assessed the impact of 5-HT on tumor growth in vivo. We first employed a subcutaneous xenograft model in nude mice inoculated with MFC GC cells (Fig. 1i). Beginning at the time of tumor cell implantation, the mice were subjected to intraperitoneal injections of 5-HT (10 mg/kg) or vehicle every two days. Consistent with our in vitro findings, 5-HT administration significantly accelerated tumor growth (Fig. 1i). Notably, treatment with the TGM2 inhibitor cystamine markedly attenuated 5-HT-driven tumor promotion (Fig. 1i). Furthermore, we validated these results using an orthotopic YTN16 cell-derived GC model^27^. Consistently, cystamine robustly suppressed 5-HT-driven tumor growth (Fig. 1j), again highlighting the critical role of TGM2 activity in serotonin-promoted tumorigenesis.

Taken together, these data demonstrate that 5-HT exerts tumor-promoting effects in GC cells, at least in part through a TGM2-dependent serotonylation mechanism.

### Chemoproteomic profiling identifies ferroptosis-related proteins as serotonylation substrates

**Scheme 1:**
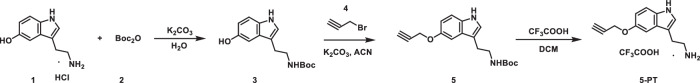
Synthesis of Compound 3: 5-Hydroxytryptamine hydrochloride (500 mg, 2.35 mmol, 1.0 equiv) was dissolved in water (9 mL). Potassium carbonate (665 mg, 4.81 mmol, 2.1 equiv) and Boc₂O (538 mg, 2.46 mmol, 1.05 equiv) were added, and the reaction mixture was stirred at room temperature overnight. Upon completion of the reaction, the mixture was extracted with dichloromethane (3 × 15 mL). The combined organic layers were dried over anhydrous Na₂SO₄, concentrated in vacuo, and purified by column chromatography to yield Compound 3 (465 mg, 85% yield).

**Fig. 2:**
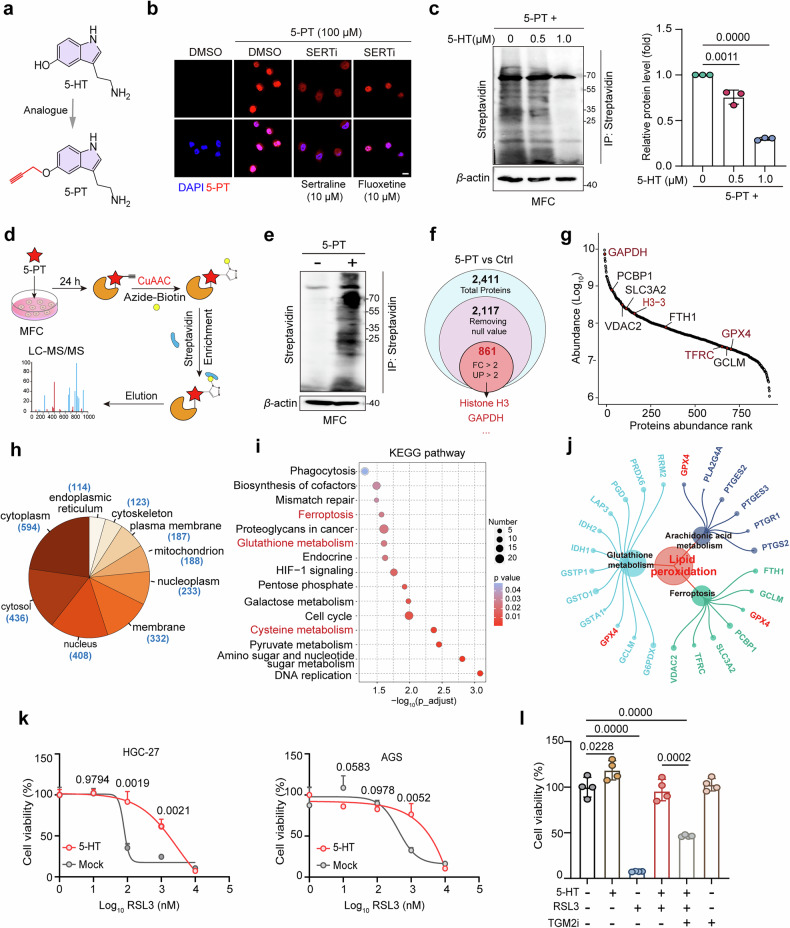
Chemoproteomic profiling revealed broad serotonylation of ferroptosis-related proteins. **a** Chemical structure of serotonin (5-HT) and its clickable probe 5-PT. **b** Representative immunofluorescence images showing 5-PT accumulation within MFC cells treated with DMSO or SERT inhibitors. Cells were incubated with 5-PT (100 μM) in the presence of a SERT inhibitor (sertraline or fluoxetine). Inhibitor concentrations are indicated. Scale bar, 10 μm. **c** Western blot analysis revealed that 5-PT labeling efficiency was reduced by 5-HT in a dose-dependent manner. MFC cells were treated with 5 PT or 5-PT + 5-HT for 24 h. Quantification is shown on the right (*n* = 4 per group). **d** Workflow for labeling substrate proteins using 5-PT via click chemistry. **e** Immunoprecipitation was performed on MFC cells incubated with or without 5-PT (100 μM) via click chemistry. **f** Venn analysis of 5-PT-based proteome in MFC cells. A stringent data filtering strategy was applied to the 5-PT-based proteomics dataset. Proteins were defined as high-confidence serotonylation candidates on the basis of a fold change (FC) > 2 and a unique peptide (UP) number > 2. **g** Rank analysis of the 5-PT-based proteomics results. Serotonylated proteins in the ferroptosis pathway are shown. **h** Subcellular locations of the serotonylated proteins. **i** KEGG pathway analys**i**s of the 861 serotonylated proteins. **j** Crosstalk between lipid metabolism and ferroptosis centered on GPX4, underscoring the central role of GPX4 in suppressing lipid peroxidation and determining ferroptosis sensitivity. **k** Relative viability of HGC-27 and AGS cells treated with 5-HT (100 μM) and various concentrations of RSL3 for 24 h (*n* = 4 per group). **l** GPX4 serotonylation suppresses ferroptosis in HGC-27 cells. The relative viability of HGC-27 cells was measured after treatment with 5-HT (10 μM), RSL3 (500 nM), and cystamine (100 μM) for 24 h (*n* = 4 per group). Data are presented as mean ± SD from two or three independent experiments. Statistical analysis was performed using ordinary one-way ANOVA (**c**, **l**) or an unpaired *t* test (**k**).

Having established the pro-tumorigenic role of protein serotonylation, we next sought to systematically identify its substrate proteins in GC cells. To this end, we employed a chemoproteomic approach using 5-PT (Scheme 1; Supplementary Fig. S2a), a clickable functional analog of 5-HT (Fig. 2a)^28^. Click chemistry-coupled immunofluorescence confirmed the efficient cellular uptake of 5-PT (Fig. 2b; Supplementary Fig. S2b, c). Moreover, treatment with a serotonin transporter (SERT) inhibitor (sertraline or fluoxetine) resulted in a pronounced reduction in the 5-PT signal (Fig. 2b), indicating the similarity of this probe to 5-HT. Furthermore, 5-PT competes with 5-HT for covalent modification of cellular proteins, thereby establishing an optimized protocol for profiling the serotonylome in GC models (Fig. 2c). In MFC cells, 5-PT labeled numerous intracellular proteins, and this labeling was efficiently outcompeted by increasing concentrations of 5-HT (Fig. 2c).

For comprehensive substrate identification, MFC cells were treated with 5-PT for 24 h and then lysed, after which the labeled proteins were enriched via click chemistry, followed by streptavidin pulldown. To minimize nonspecific binding, the beads were thoroughly washed with 8 M urea before the enriched proteins were analyzed by 4D label-free mass spectrometry (Fig. 2d)^29^. Western blot analysis confirmed the efficient labeling and enrichment of serotonylated proteins (Fig. 2e). After removing null values and applying strict cutoffs of a fold change (FC) > 2 and a unique peptide (UP) number >2 861 proteins specifically enriched in the 5-PT-treated group were identified by LC-MS/MS analysis (Fig. 2f; Supplementary Table S2). Notably, this approach identified previously reported serotonylation targets, such as GAPDH^23^ and histone H3^18^, validating the robustness and specificity of our methodology (Fig. 2f, g). Subcellular localization analysis revealed that more than half of these serotonylated proteins were cytoplasmic (Fig. 2h), which led us to subsequently focus on this compartment.

To elucidate the biological functions of the identified serotonylated proteins, we performed Gene Ontology (GO) analysis (Supplementary Fig. S2d), which indicated that serotonylation may influence protein‒protein interactions. Biological process (BP) analysis revealed significant enrichment of proteins involved in intracellular protein transport and metabolic regulation (Supplementary Fig. S2e). Kyoto Encyclopedia of Genes and Genomes (KEGG) analysis revealed that these serotonylated proteins are involved mainly in cellular metabolism, DNA replication, DNA repair, the cell cycle and ferroptosis (Fig. 2i). Notably, integrated pathway mapping placed lipid peroxidation as a central node of a network involving glutamine metabolism, arachidonic acid pathways, and ferroptosis, with GPX4 as a shared component (Fig. 2j).

Given the established link between phospholipid peroxidation, ferroptosis, and tumor growth^30–32^, we further investigated how protein serotonylation regulates ferroptosis in the context of GC. To this end, we examined whether 5-HT directly influences ferroptosis. Treatment with 5-HT significantly attenuated the RSL3-induced ferroptosis of both HGC-27 and AGS cells (Fig. 2k). More importantly, this protective effect was effectively abolished by the inhibition of TGM2 (Fig. 2l), suggesting that the serotonylation of key proteins in the ferroptosis pathway confers resistance.

### GPX4 is a bona fide TGM2 serotonylation substrate in GC cells

**Fig. 3:**
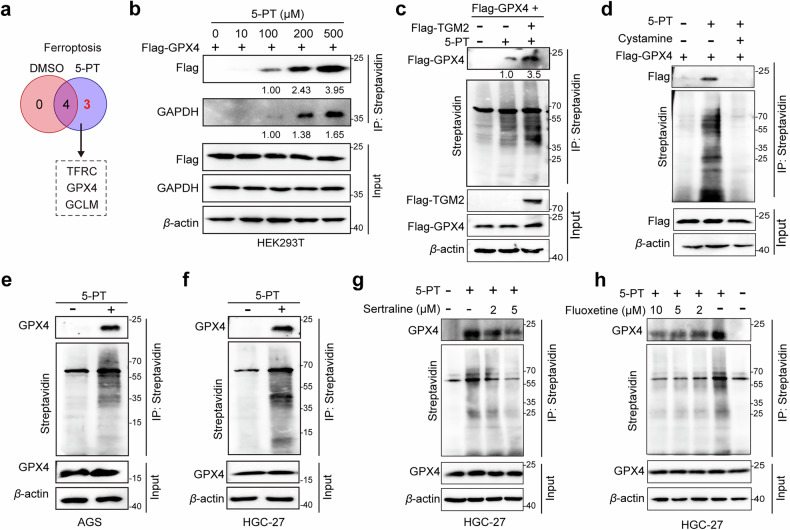
GPX4 is a bona fide TGM2 serotonylation substrate in GC cells. **a** Venn diagram comparing ferroptosis-related proteins detected in DMSO- and 5-PT-treated samples. Proteins unique to the 5-PT condition include TFRC, GPX4, and GCLM. **b** Western blotting of the serotonylation of Flag-GPX4 in HEK293T cells. GAPDH was used as a reference. **c**, **d** Western blot analysis of GPX4 serotonylation in the presence of TGM2. **c** TGM2 overexpression, **d** with or without the TGM2i cystamine (4 mM). **e** 5-PT-based click chemistry assay for the detection of endogenous GPX4 serotonylation in AGS cells. **f** 5-PT-based click chemistry assay for the detection of endogenous GPX4 serotonylation in HGC-27 cells. **g** 5-PT-based click chemistry assay demonstrating that GPX4 serotonylation in GC cells is inhibited by a SERT inhibitor (sertraline). **h** 5-PT-based click chemistry assay demonstrating that GPX4 serotonylation in GC cells is inhibited by a SERT inhibitor (fluoxetine).

Given the ability of 5-HT-mediated protein serotonylation to confer ferroptosis resistance, we sought to identify specific serotonylation targets within the ferroptosis pathway. Our LC-MS/MS analysis revealed that three ferroptosis-associated proteins, namely, TFRC^33^, GPX4^34^, and GCLM^35^, were specifically enriched in the 5-PT-labeled fraction (Fig. 3a). Subsequent 5-PT-based serotonylation assays confirmed clear, dose-dependent modification of GPX4 along with the known serotonylation substrates GAPDH^23^ (Fig. 3b) and TFRC (Supplementary Fig. S3a). Given the central role of GPX4 in lipid metabolism and ferroptosis regulation, as well as the observation that TGM2-mediated serotonylation promotes ferroptosis resistance, we performed subsequent mechanistic studies on GPX4.

To experimentally validate GPX4 as a serotonylation target, we performed a series of cellular assays. In HEK293T cells expressing Flag-GPX4, treatment with increasing concentrations of 5-PT resulted in dose-dependent covalent labeling of exogenously expressed GPX4 (Fig. 3b). Ectopic expression of TGM2 further increased Flag-GPX4 serotonylation in a dose-dependent manner (Fig. 3c), whereas inhibition of TGM2 with cystamine completely abolished this modification (Fig. 3d). In addition, endogenous GPX4 serotonylation was reproducibly detected in both AGS (Fig. 3e) and HGC-27 (Fig. 3f) GC cells. Furthermore, consistent with SERT being the primary transporter for serotonin uptake (Fig. 2c), pharmacological inhibition of SERT with sertraline or fluoxetine reduced GPX4 serotonylation in a dose-dependent manner (Fig. 3g, h). Finally, we assessed GPX4 serotonylation levels under basal conditions, upon ferroptosis induction (with RSL3), and under ferroptosis inhibition (with ferrostatin-1). The results showed that GPX4 serotonylation remained stable across these conditions (Supplementary Fig. S3b).

Collectively, these results indicate that GPX4 is a bona fide TGM2 serotonylation substrate, representing a mechanism that promotes GC cell proliferation through the suppression of ferroptosis.

### GPX4 is specifically serotonylated at residues Gln55 and Gln77

Having established that TGM2 mediates the serotonylation of GPX4, we next aimed to identify the specific glutamine (Gln/Q) residues targeted for modification^36,37^. Sequence alignment revealed five evolutionarily conserved glutamine residues in GPX4, Q45, Q55, Q77, Q81, and Q123 (Supplementary Fig. S4a), the spatial localization of which suggests accessibility for TGM2-mediated modification (Supplementary Fig. S4b). To identify the sites of covalent 5-HT attachment, we systematically mutated each glutamine to alanine (A) and assessed serotonylation using a 5-PT-based assay in HEK293T cells (Supplementary Fig. S4c). Cells were transfected with wild-type (WT) or mutant GPX4 plasmids for 48 h, lysed, and incubated with 5-PT, followed by detection via copper-catalyzed click chemistry. Notably, mutations at Q55, Q77, and Q123 significantly impaired GPX4 serotonylation, whereas mutations at Q45 and Q81 had minimal effects (Supplementary Fig. S4d, e).

**Fig. 4:**
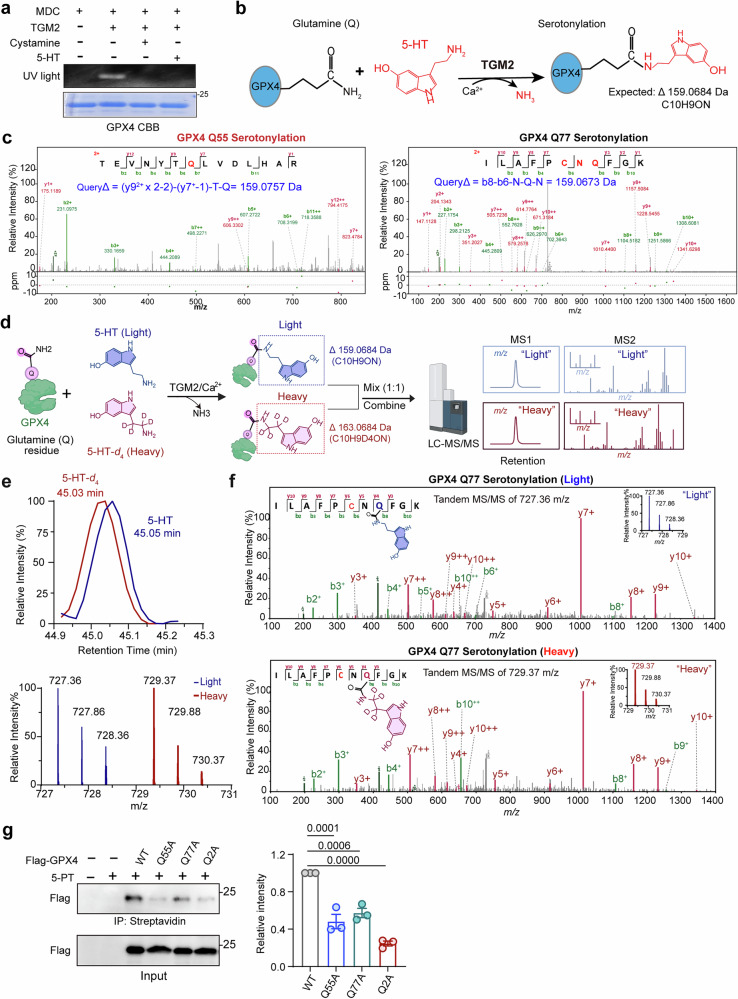
GPX4 is specifically serotonylated at the Gln55 and Gln77 residues. **a** TGM2-dependent transamidation signals in recombinant GPX4 (monitored via monodansylcadaverine fluorescence) that were abolished by either cystamine inhibition or 5-HT competition. **b** Schematic illustration of the TGM2-dependent transamidation of glutamine residues in substrate proteins. **c** MS/MS spectra of the serotonylated GPX4 peptide (GPX4ser: Q55, Q77) derived from GPX4 (in vitro). The b ion refers to the N-terminal portion of the peptide, and the y ion indicates to the C-terminal portion. **d** SILAC experimental workflow for GPX4 serotonylation detection by LC-MS/MS using 5-HT and 5-HT-*d*_4_. TGM2 catalyzes the transamidation of glutamine residues in the GPX4 protein. **e** Chromatographic overlay of 5-HT and 5-HT-*d*_4_, demonstrating nearly identical retention times or 45.03 min and 45.05 min, respectively (top). MS analysis of 5-HT and 5-HT-*d*_4_ revealed distinct isotopic peaks. Mass spectra of Light, with m/z of 727.36 and Heavy, with m/z of 729.37 m/z for 5-HT and 5-HT-*d*_4_, respectively, illustrating the clear separation between the light and heavy isotopic labels and confirming the use of deuterium-labeled serotonin for dual validation (bottom). **f** Tandem MS/MS spectrum of the peak at m/z 727.36 (5-HT Light) showing the fragmentation pattern of GPX4 at the serotonylated site, confirming the presence of 5-HT modification (top). Tandem MS/MS spectrum of the peak at m/z 729.37 (5-HT-*d*_4_ Heavy) showing the corresponding fragmentation pattern, confirming the site-specific serotonylation of GPX4 (bottom). **g** Immunoblot and streptavidin pull-down analysis of Flag-GPX4 with various mutations (Q54A, Q77A, and Q2A) in HEK293T cells treated with 5-PT, confirming the loss of serotonylation at specific sites. Quantification is shown on the right (mean ± SEM; ordinary one-way ANOVA, *n* = 3).

We subsequently purified the recombinant GPX4 protein and performed an in vitro monodansylcadaverine (MDC) assay to confirm TGM2-dependent transamidation. TGM2 catalyzed the covalent attachment of the auto-fluorescent MDC probe to GPX4, whereas treatment with cystamine or an excess of 5-HT markedly reduced this reaction (Fig. 4a), confirming that TGM2 directly mediates GPX4 serotonylation in vitro. To precisely map the modification sites, we analyzed the in vitro serotonylated recombinant GPX4 protein by LC-MS/MS. By covalently attaching a serotonin group to a glutamine residue, serotonylation results in a protein with characteristic mass shift of +159.0684 Da (serotonin minus NH_3_; Fig. 4b). Consistent with our mutational analysis, strong diagnostic mass shifts corresponding to serotonylation were detected specifically at Gln55 (+159.0757 Da; Fig. 4c, left) and Gln77 (+159.0673 Da; Fig. 4c, right). In contrast, only a negligible shift was observed at Gln123 (−0.0001 Da; Supplementary Fig. S4f). These data establish Gln55 and Gln77 as the principal sites of GPX4 serotonylation.

To validate these findings and improve the signal-to-noise ratio, we conducted stable isotope labeling by amino acids in cell culture (SILAC)-based dual labeling experiments using 5-HT (light) and deuterated 5-HT-*d*_4_ (heavy). After independent TGM2-catalyzed serotonylation reactions with each isotopologue, the samples were combined at a 1:1 ratio, desalted, and analyzed by LC-MS/MS (Fig. 4d). The two forms eluted chromatographically at nearly identical retention times, and their MS1 isotopic envelopes showed the expected 4 Da mass difference (Fig. 4e). Peptide identification was performed for pairs containing light- or heavy-modified residues, and isotopic intensity ratios (H/L) were calculated. Notably, a strong serotonylation signal was specifically detected at Gln77 with both isotopologues (Fig. 4f). Serotonylation at Gln55 was also clearly identified in the light-labeled sample (Supplementary Fig. S4g), whereas no modification was observed at Gln123 (Supplementary Fig. S4h).

In addition, we generated a loss-of-function mutant by substituting both Q55 and Q77 with alanine (hereafter denoted GPX4-2QA, GPX4^2A^) and assessed serotonylation using a 5-PT-based assay in HEK293T cells (Fig. 4g). The results revealed that mutations at Q55 and Q77 significantly impaired GPX4 serotonylation (Fig. 4g). Collectively, these data demonstrate that GPX4 is predominantly serotonylated at the Gln55 and Gln77 residues.

### Serotonylation stabilizes GPX4 by impeding its ubiquitin-mediated proteasomal degradation

**Fig. 5:**
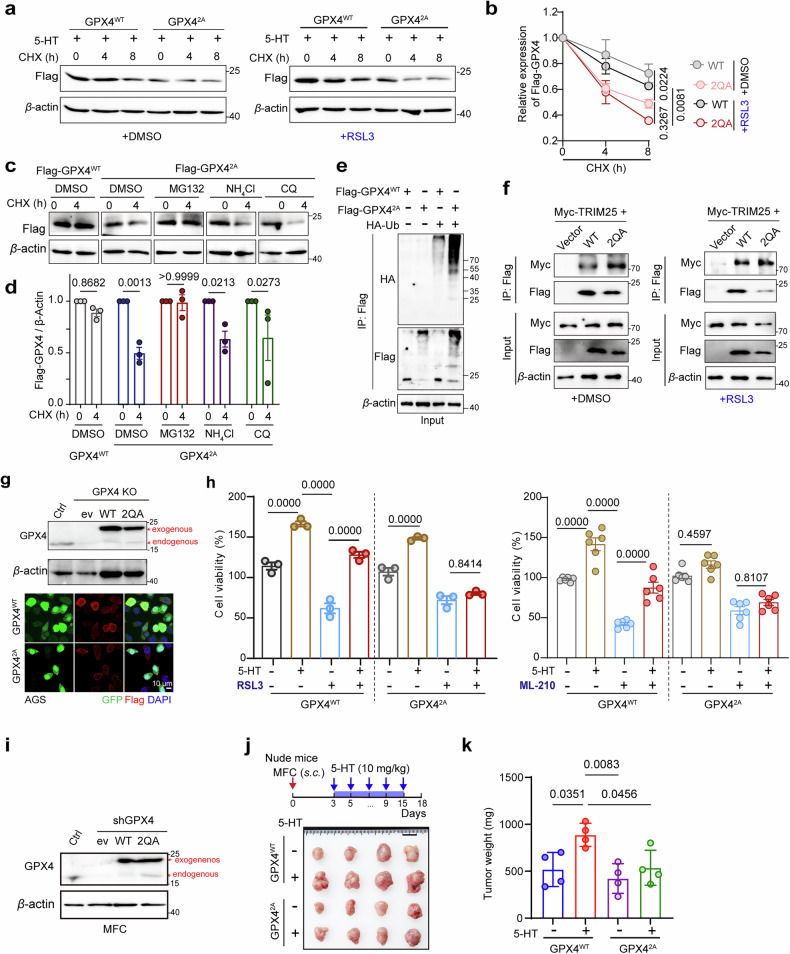
Serotonylation stabilizes the GPX4 protein to confer ferroptosis resistance. **a** HEK293T cells transfected with GPX4^WT^ or GPX4^2A^ were treated with 5-HT (100 μM) and analyzed using CHX (50 μM) chase experiments at different time points. CHX chase assays were performed to analyze the protein stability of GPX4^WT^ and GPX4^2A^ in the absence (left) or presence (right) of a ferroptosis inducer (RSL3). **b** Quantification of GPX4 protein levels (Flag) from Panel (**a**). **c** Western blot showing the stability of HEK293T cells transfected with GPX4^WT^ or GPX4^2A^. Cells were treated with 50 μg/mL CHX for the indicated duration (0 h or 4 h) in the absence or presence of the proteasome inhibitor MG132 (10 μM), the lysosomal acidification inhibitor NH₄Cl, or the autophagy inhibitor chloroquine (CQ). **d** Relative levels of Flag-GPX4 and its mutants in the CHX chase assay (**c**). **e** HEK293T cells transfected with WT or the GPX4^2A^ mutant were co-transfected with HA-Ub for 24 h before being harvested. Ubiquitinated GPX4 was detected with an anti-Flag antibody. **f** Myc-TRIM25 overexpression in HEK293T cells co-transfected with Flag-tagged GPX4^WT^ or GPX4^2A^ mutants. In the absence (left) or presence (right) of RSL3, the interaction between TRIM25 and GPX4 was detected by immunoprecipitation (IP) with an anti-Flag antibody and western blotting for Myc. **g** Western blot validation of exogenous GPX4 transfection efficiency in GPX4-KO AGS cells. Immunofluorescence images (bottom) of AGS cells stably expressing GPX4^WT^ or GPX4^2A^. Cells were stained with DAPI (blue), GFP (green), and Flag-GPX4 (red). Scale bars, 10 μm. **h** The relative viability of AGS cells transfected with GPX4^WT^ or GPX4^2A^ was measured following treatment with 5-HT (10 μM) and RSL3 (500 nM) or ML-210 (5 μM) for 24 h (*n* = 3–5). **i** Western blot analysis validating the expression of exogenous Flag-tagged GPX4 (WT) and the Q2A mutant in GPX4-silenced MFC cells (shGPX4). The specific expression of exogenous and endogenous proteins was detected using an anti-GPX4 antibody. **j** Schematic diagram of the workflow for analyzing subcutaneous MFC tumors (5 × 10^6^ cells) in nude mice treated with or without 5-HT (10 mg/kg) for 12 days. Insert: Representative images of subcutaneous tumors are shown on the bottom. **k** Weights of subcutaneous MFC tumors in nude mice (*n* = 4 per group). Data are presented as mean ± SD from two or three independent experiments. Statistical analysis was performed using two-way ANOVA (**b**, **d**) or ordinary one-way ANOVA (**h**, **k**).

To investigate the functional impact of GPX4 serotonylation, we first examined whether 5-HT treatment affects GPX4 protein stability. Indeed, treatment with 5-HT increased GPX4 levels in a dose-dependent manner in both HGC-27 and AGS cells (Supplementary Fig. S5a). We then tested whether serotonylation is required for this effect. To this end, we compared the protein stability of WT GPX4 and the serotonylation-deficient GPX4-2QA mutant using cycloheximide (CHX) chase assays. 5-HT significantly prolonged the half-life of WT GPX4 but not the GPX4-2QA mutant (Fig. 5a), indicating that serotonylation at Q55 and Q77 is required for the 5-HT-mediated stabilization of GPX4. Furthermore, this stabilization effect persisted upon treatment with the ferroptosis inducers RSL3 (Fig. 5b) and ML-210 (Supplementary Fig. S5b). In contrast, the GPX4-2QA mutant did not stabilize under any of these conditions (Fig. 5b; Supplementary Fig. S5b).

We next sought to identify the downstream route responsible for GPX4 degradation. To this end, both WT-GPX4- and GPX4-2QA-expressing cells were treated with CHX in combination with the proteasome inhibitor MG132, the lysosomal inhibitor NH₄Cl, or the autophagy inhibitor chloroquine (CQ) and harvested for western blot analysis 4 h later (Fig. 5c, d). As expected, the GPX4-2QA mutant degraded more rapidly than WT GPX4 did. Importantly, co-treatment with MG132, but not with NH₄Cl or CQ, completely blocked the degradation of GPX4-2QA (Fig. 5c, d), indicating a proteasome-dependent process. Similarly, ubiquitination assays performed by co-expressing HA-ubiquitin revealed markedly increased poly-ubiquitination of GPX4-2QA compared with that of WT GPX4 (Fig. 5e). These findings led us to speculate that serotonylation protects GPX4 by interfering with its recognition by an E3 ubiquitin ligase. In this context, we focused on TRIM25, which has been reported to mediate the K48-linked ubiquitination and proteasomal degradation of GPX4^38,39^. Co-immunoprecipitation (co-IP) experiments confirmed a robust interaction between TRIM25 and GPX4, and this interaction was markedly stronger with GPX4-2QA than with WT GPX4 (Fig. 5f, left). Moreover, the TRIM25–GPX4-2QA association was further enhanced under ferroptosis-inducing conditions (Fig. 5f, right), supporting the notion that serotonylation attenuates the TRIM25-mediated recognition and ubiquitination of GPX4, thereby increasing its stability.

Taken together, these data indicate that serotonylation prevents the proteasomal degradation of GPX4 by attenuating its recognition and ubiquitination by TRIM25.

### GPX4 serotonylation drives ferroptosis resistance to support tumor growth

To definitively assess the role of GPX4 serotonylation in GC cell resistance to ferroptosis, we generated endogenous GPX4-knockout AGS cells and reconstituted them with either WT GPX4 (GPX4^WT^) or the serotonylation-defective mutant GPX4^2A^ (Fig. 5g). In this isogenic system, 5-HT robustly promoted cell proliferation and protected against ferroptosis induced by either RSL3 or ML-210, specifically in GPX4^WT^ cells but not in GPX4^2A^ cells (Fig. 5h; Supplementary Fig. S5c). Similar results were obtained in GPX4-knockout HGC-27 cells reconstituted with GPX4^WT^ or GPX4^2A^ (Supplementary Fig. S5d–f), confirming the general requirement of GPX4 serotonylation for 5-HT-mediated ferroptosis resistance.

Finally, to establish in vivo relevance, we performed xenograft experiments in which GPX4-knockout MFC cells were reconstituted with similar levels of GPX4^WT^ or GPX4^2A^ (Fig. 5i). Systemic serotonin administration significantly promoted the growth of GPX4^WT^ tumors but not GPX4^2A^ tumors (Fig. 5j, k), providing direct in vivo evidence that GPX4 serotonylation promotes tumor growth by conferring ferroptosis resistance.

Collectively, these data, including multiple ferroptosis inducers, rigorous genetic rescue models, and in vivo validation, establish GPX4 serotonylation as a key mechanism through which serotonin drives ferroptosis resistance and tumor progression in the context of GC.

### GPX4 serotonylation is correlated with tumor progression and poor prognosis

**Fig. 6:**
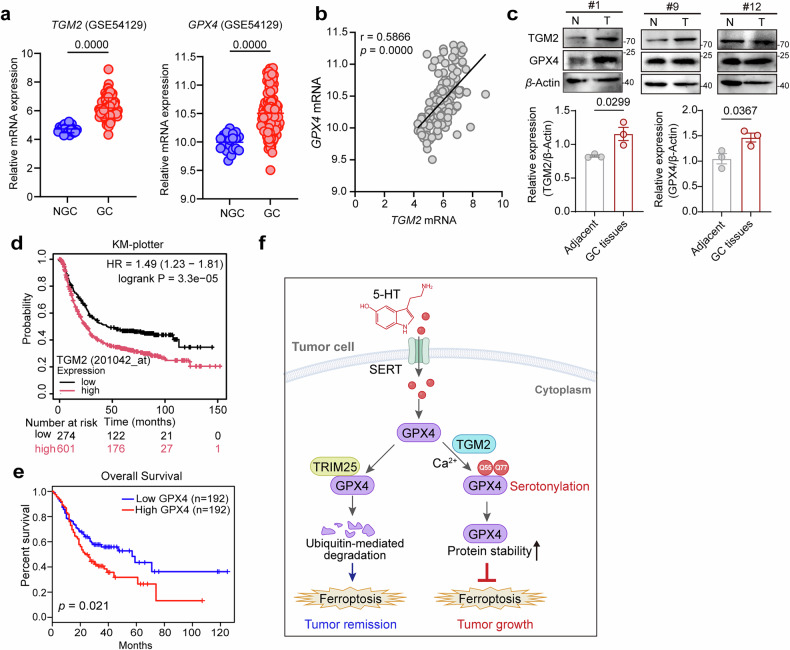
Clinical correlation analysis of the TGM2-GPX4ser axis in tumor progression. **a** RNA-seq analysis was conducted to compare the relative expression levels of TGM2 and GPX4 between healthy controls (NGC) and GC patients using data from the GSE54129 dataset. **b** Correlation between TGM2 and GPX4 mRNA levels in the GSE54129 dataset. The Pearson correlation (*r*) and *P* value are shown. **c** Representative western blot analysis of TGM2 and GPX4 expression levels in GC versus adjacent normal tissues (*n* = 3; unpaired *t* test). **d** Kaplan‒Meier survival analysis of GC patients with low or high TGM2 expression was performed with KM plotter. **e** Overall survival (OS) of GC patients stratified by GPX4 expression level (*P* = 0.021), as analyzed using the online tool GEPIA2. **f** Graphical abstract: TGM2-catalyzed serotonylation of GPX4 at the Q55 and Q77 residues stabilizes the GPX4 protein and protects it from TRIM25-dependent proteasomal degradation, conferring tumor cell resistance to ferroptosis and resulting in cell proliferation and tumor growth. TGM2 inhibition can abolish these effects, providing a potential direction for targeted GC therapy.

We further evaluated the clinical relevance of the TGM2-GPX4 serotonylation axis in GC. Analysis of the RNA sequencing (RNA-seq) data from a GEO dataset (GSE54129) revealed that among all the TGM isoforms, TGM2 was the most highly expressed in the GC samples (Fig. 6a). Both TGM2 and GPX4 were significantly upregulated in tumor tissues compared with their expression in noncancerous gastric tissues (Fig. 6a). Moreover, correlation analysis revealed a strong positive correlation between TGM2 and GPX4 mRNA levels in the tumor microenvironment (*r* = 0.5886; *P* < 0.0001; Fig. 6b). To experimentally validate these findings, we examined TGM2 and GPX4 protein levels in seven paired clinical GC specimens. In approximately half of the patients (3/7), compared with the adjacent normal mucosa, tumor tissues presented markedly elevated GPX4 expression, which was accompanied by a concurrent increase in TGM2 levels (Fig. 6c). These results suggest that TGM2-mediated serotonylation of GPX4 occurs in vivo and may contribute to GC progression. Survival analysis revealed that high expression of either TGM2 (Fig. 6d) or GPX4 (Fig. 6e) was associated with significantly poorer overall survival in patients with GC.

Collectively, our data establish that TGM2-mediated GPX4 serotonylation is a clinically relevant mechanism that promotes ferroptosis resistance and tumor proliferation. These findings highlight the TGM2-GPX4ser axis as a potential diagnostic and prognostic biomarker in patients with gastrointestinal cancers, with important implications for future therapeutic strategies.

## Discussion

Serotonin, traditionally known as a key regulator of gastrointestinal motility, has recently emerged as a multifunctional modulator of cancer progression through both receptor-mediated signaling and post-translational serotonylation. While previous studies have highlighted the importance of HTR2B-dependent signaling in GC pathogenesis, our work establishes that TGM2-catalyzed serotonylation is a parallel and indispensable pathway in this context. By systematically identifying GPX4 as a key substrate of serotonylation, we revealed a previously unrecognized mechanism through which 5-HT increases ferroptosis resistance and promotes tumor growth (Fig. 6f). These findings not only broaden the scope of known serotonylation targets but also provide a direct molecular link between neural signaling and redox homeostasis in cancer. Our integrated model positions serotonylation as a complementary axis to receptor-mediated signaling, suggesting that effective therapeutic targeting of the serotonin pathway in GC cells may require dual inhibition of both 5-HT receptors and this TGM2-mediated modification (Fig. 6f).

Neurotransmitter-driven protein modifications, such as serotonylation, dopaminylation, and histaminylation, are being increasingly recognized as emerging regulatory paradigms in cancer biology^26,40–42^. We first performed untargeted metabolomics to elucidate the global plasma metabolite profile of GC patients in comparison to those of healthy donors. This analysis generated a list of significantly altered metabolites (Supplementary Table S1). Among the most increased metabolites, we prioritized 5-HT for further investigation on the basis of the following: (1) the increasing recognition of neural–cancer crosstalk as a hallmark of cancer and (2) the growing interest in serotonylation as a novel, receptor-independent signaling mechanism in tumor biology. Our study demonstrates that TGM2-catalyzed serotonylation serves as a key mechanism through which serotonin promotes GC progression. Using chemoproteomic profiling, we then applied strict cutoffs of a fold change (FC) > 2 and a unique peptide (UP) number > 2 to define high-confidence serotonylated candidates, yielding a total of 861 proteins with putative serotonylation sites. Importantly, integrated pathway mapping revealed that lipid peroxidation and its related pathways, including glutamine metabolism, arachidonic acid metabolism, and ferroptosis, formed a central network. Within this network, GPX4 emerged as a shared node connecting multiple pathways. Given the established link between phospholipid peroxidation, ferroptosis, and tumor growth^30–32^, we prioritized investigating how GPX4 serotonylation regulates ferroptosis to support GC proliferation. This modification occurs primarily at the Gln55 and Gln77 residues, increases GPX4 protein stability, and confers resistance to ferroptosis, an iron-dependent form of regulated cell death. The consequent suppression of ferroptosis permits sustained tumor proliferation under oxidative stress. Importantly, pharmacological inhibition of TGM2 with cystamine abolished serotonin-driven tumor growth, underscoring the functional relevance of this pathway. These findings are further contextualized by recent work from Zheng et al.^43^, whose mass spectrometry data suggest that GPX4 may also undergo dopaminylation, indicating that monoamine-based modifications constitute a broader layer of post-translational regulation in cancer. Together, our results not only highlight serotonylation as a therapeutically targetable mechanism in GC but also position monoamine-mediated protein modifications as a promising frontier for anticancer drug development.

GPX4 is known to be regulated by various post-translational modifications (PTMs), including phosphorylation^44^, ubiquitination^44^, succinylation^45^, acetylation^44^, and methylation^46^, that critically influence its stability, catalytic activity, and subcellular localization. These PTMs constitute key regulatory nodes in oxidative stress responses and ferroptosis control. Recent studies have further expanded this landscape; for example, palmitoylation at Cys66 and Cys75, which is catalyzed by ZDHHC enzymes, increases GPX4 membrane association and stability, thereby promoting ferroptosis resistance. The inhibition of palmitoylation sensitizes cancer cells to ferroptosis, underscoring its therapeutic potential^47,48^. In this context, our work identifies serotonylation as a novel regulatory PTM of GPX4. We show that TGM2-catalyzed serotonylation at Gln55 and Gln77 increases GPX4 stability and suppresses ferroptosis, providing a mechanistic basis for serotonin-driven gastric tumorigenesis. In this context, the E3 ligase TRIM25 has been reported to promote the K48-linked ubiquitination and proteasomal degradation of GPX4^38,39^. We found that serotonylation of GPX4 prevents its recruitment to TRIM25, leading to a longer half-life of the GPX4 protein. In support of this notion, our co-IP assays revealed a markedly stronger interaction between TRIM25 and GPX4^2A^ than between TRIM25 and WT GPX4.

Crosstalk between neurotransmitter signaling and GPX4-mediated ferroptosis defense represents a previously unrecognized adaptive mechanism utilized by cancer cells under metabolic stress. Importantly, survival analyses support the clinical relevance of the TGM2-GPX4 axis, positioning GPX4 serotonylation as both a prognostic biomarker and a promising therapeutic target. Together, these findings not only expand the repertoire of GPX4 PTMs but also suggest that targeting serotonylation may offer a novel strategy to overcome ferroptosis resistance in gastric and other gastrointestinal cancers.

In conclusion, our study reveals that serotonin promotes gastric tumorigenesis via receptor-independent mechanisms, with TGM2-mediated serotonylation of GPX4 representing a previously unidentified pathway. This covalent modification increases GPX4 stability and suppresses ferroptosis, thereby conferring a critical survival advantage to cancer cells. Our results not only advance the understanding of neurotransmitter-driven cancer progression but also establish serotonylation as a promising therapeutic target, opening new avenues for targeted intervention in GC.

### Limitations and future directions

Although our study identifies GPX4 serotonylation as a potential diagnostic and prognostic biomarker in gastrointestinal cancers, several limitations should be acknowledged. First, the development of a specific antibody would allow more accurate detection and validation of this modification in clinical specimens. Second, the broader impact of serotonylation on the tumor immune microenvironment remains unexplored and represents an important direction for future research, particularly for TFRC, which we validated as a bona fide serotonylation target. Third, while our cellular and animal models provide mechanistic insights, the physiological relevance of GPX4 serotonylation in the context of human GC requires further validation in larger clinical cohorts. Fourth, the precise effect of serotonylation on GPX4 enzymatic activity remains to be elucidated. Finally, the functional contributions of non-GPX4 serotonylation targets to gastric tumorigenesis warrant systematic investigation. Addressing these questions will not only improve our understanding of serotonin-driven tumor progression but also facilitate therapeutic targeting of the TGM2-GPX4 serotonylation axis in gastrointestinal cancer patients.

## Materials and methods

### Cell lines

The HEK293T, AGS, and HGC-27 cell lines were obtained from the cell library of the Chinese Academy of Sciences (Shanghai, China)^49^. YTN16 cells^27^ were a gift from Prof. Sachiyo Nomura (The University of Tokyo). MFC cells were purchased from the National Infrastructure of Cell Line Resource (Beijing, China)^50^. HEK293T and MFC cells were cultured in DMEM (Basal Media, L110KJ), and AGS and HGC-27 cells were cultured in RPMI 1640 medium (Basal Media, L210KJ) supplemented with 10% FBS (Vital Cell Bioscience, C04001-500) and 1% penicillin and streptomycin (Basal Media, S110JV) at 37 °C with 5% CO_2_.

### Plasmids

Full-length TGM2 and GPX4 as well as their corresponding mutants were all cloned and inserted into pcDNA3.1 or pCDH vectors. For protein expression in *Escherichia coli*, full-length GPX4 and TGM2 were cloned and inserted into modified pET28a vectors with the indicated tags. All the constructs were verified by sequencing.

### Mouse tumor models

Nude mice (males, 4–5 weeks old) and C57BL/6 mice (males, 6–8 weeks old) were housed in ventilated cages under specific pathogen-free conditions and given ad libitum access to food and water. MFC cells (5 × 10^6^) or YTN16 cells (1 × 10^7^) were suspended in 0.1 mL of PBS and subcutaneously injected into the left abdomen of the mice. Tumor growth was monitored with an electronic caliper by measuring tumor size every 2 days. The tumor volume was calculated as (1/2) × length × width^2^. All animal experiments were conducted in accordance with the guidelines for the ethical use of animals in research (Approval Number: SHDSYY-2023-P0011).

### Cell transfection

HEK293T cells were plated in DMEM containing 10% FBS, the target plasmids were diluted with Opti-MEM (Gibco), and then DNA and polyethylenimine hydrochloride (PEI; Polysciences, 31985–070) were mixed at a ratio of 1:3. The DNA‒PEI mixture was then incubated at room temperature for 30 min before being added dropwise to the HEK293T cell dishes. After 24–48 h, the cells were collected and treated for subsequent assays.

### Western blotting

Cells were collected and treated with pre-cooled RIPA lysis buffer (50 mM Tris HCl, pH 8.0, 1% Triton X-100, 0.1% SDS, 150 mM NaCl, 0.5% sodium deoxycholate and 1 mM PMSF), placed on ice for 15 min and then heated to 99 °C for 10 min with 5× SDS loading buffer. Next, the samples were separated on SDS‒PAGE gels, transferred to a PVDF membrane (Amersham), and further incubated with the indicated primary antibodies. The primary antibodies and dilutions used for western blotting were as follows: anti-Flag (Sigma‒Aldrich, F3165, 1:4000), anti-GPX4 (Abcam, ab125066, 1:5000), anti-TGM2 (ABclonal, A21184, 1:2000), anti-Myc (Santa Cruz, 9E10): sc-40, 1:1000), anti-TFRC (CST, #13113), GAPDH (Yeasen, 30201ES20, 1:2000), anti-*β*-actin (Sigma‒Aldrich, A2228, 1:4000), and anti-HRP-streptavidin (Yeasen, 35105ES60, 1:8000). The immunoblotting images were scanned using a chemiluminescence imaging and analysis system (Sage Creation Science).

### Cell viability and colony formation assays

Cell viability was assessed using a Cell Counting Kit-8 (Yeasen, 40203ES60) and CellTiter (Beyotime, C0068S). Cells were plated at 2000 cells per well in 96-well plates and starved in serum-free medium for 12 h. Subsequently, the cells were incubated with the indicated concentrations of 5-HT (Sigma‒Aldrich, H9523), RSL3 (Selleck, S8155), ML-210 (MCE, HY-100003), SB 204741 (MCE, HY-103153), or ERW1041E (Sigma‒Aldrich, 509522) for 24 h. For the colony formation assay, the cells were plated at 1000 cells per well in a 6-well plate. After 7–14 days of incubation with or without 5-HT, the cell colonies were stained with 0.5% crystal violet and subjected to statistical analysis. The results were analyzed and plotted using GraphPad Prism 9 software.

### Generation of stable cell lines and gene perturbation assays

Stable cell lines were generated using a lentiviral transduction system. Briefly, lentiviral expression vectors were co-transfected with the packaging plasmids psPAX2 and pMD2.G into HEK293T cells at a ratio of 4:3:1. After 48 h, the viral supernatants were collected and filtered through a 0.45 μm membrane filter. Target cells were transduced with the filtered supernatants in the presence of 8 μg/mL polybrene for 48 h, followed by selection with 2 μg/mL puromycin or sorting by flow cytometry. The knockdown or knockout efficiency was verified by western blotting.

For GPX4 knockdown and knockout, lentiviral vectors encoding GPX4-specific shRNAs or sgRNAs were used to infect YTN16, MFC, AGS, or HGC-27 cells. Stable cell lines were established by puromycin selection or flow cytometry sorting. For GPX4 knockdown, mouse GPX4-specific shRNAs with the following target sequences were used: shRNA-1#, ATGCCATCAAATGGAACTTTA; and shRNA-2#, ACAGCAAGATCTGTGTAAATG. A non-targeting shRNA served as the control. These constructs were kindly provided by Xiangdong Cheng^51^. For GPX4 knockout, human GPX4-specific sgRNAs were designed with the following target sequences: sgRNA-1#, CGTGTGCATCGTCACCAACG; and sgRNA-2#, AGAGATCAAAGAGTTCGCCG. For GPX4 overexpression, GPX4-knockdown YTN16 and MFC cells, as well as GPX4-knockout AGS and HGC-27 cells, were transduced with lentiviral vectors encoding WT GPX4 or GPX4 mutant constructs (Q55A and Q77A), followed by selection to generate stable cell lines. For TGM2 silencing assays, cells were transiently transfected with siRNAs targeting human TGM2 were synthesized by Sangon Biotech (Shanghai, China). The target sequences were as follows: TGM2 siRNA-1#, GAGCUGGUCUUAGAGAGGUGU; and TGM2 siRNA-2#, GCAACCUUCUCAUCGAGUACU. A non-targeting siRNA (GAUGGCAUGGCGGAGUGAUUU) was used as a negative control.

### Detailed 5-PT synthetic procedure

The detailed synthetic procedure and full chemical characterization of 5-PT are shown in Scheme 1, as previously described^18,28^.

Synthesis of Compound 5: Compound 3 (250 mg, 0.9 mmol, 1.0 equiv) was dissolved in anhydrous acetonitrile (8 mL), and potassium carbonate (248 mg, 1.8 mmol, 2.0 equiv) and Compound 4 (114 mg, 0.96 mmol, 1.06 equiv) were added. The mixture was heated to reflux for 5 h and then suction-filtered, after which the filtrate was concentrated in vacuo. Purification by column chromatography yielded Compound 5 (198 mg, 79% yield).

Synthesis of 5-PT: Compound 5 (200 mg, 0.64 mmol, 1.0 equiv) was dissolved in dichloromethane (4 mL), treated with trifluoroacetic acid (1 mL), and stirred at room temperature for 1 h. After the reaction was complete, the mixture was concentrated in vacuo and purified by reversed-phase preparative HPLC to afford 5-PT (120 mg, 75% yield). As shown in Supplementary Fig. S2a, 1H NMR (600 MHz, DMSO-*d*_6_) δ 10.91–10.88 (m, 1H), 7.95 (s, 3H), 7.28 (d, *J* = 8.7 Hz, 1H), 7.21 (d, *J* = 2.4 Hz, 1H), 7.13 (d, *J* = 2.4 Hz, 1H), 6.80 (dd, *J* = 8.7, 2.4 Hz, 1H), 4.77 (d, *J* = 2.4 Hz, 2H), 3.49 (d, *J* = 2.5 Hz, 1H), 3.08 (t, *J* = 7.7 Hz, 2H), 2.95 (t, *J* = 7.8 Hz, 2H). HRMS (ESI, positive) m/z calculated for C_13_H_14_N_2_O 214.1106, found [M + H]^+^ 215.1199.

### Click chemistry detection of protein serotonylation

Click chemistry was used to detect serotonylated proteins in this study^20^. To prepare LC-MS/MS samples, MFC cells were incubated with 5-PT (100 μM) for 24 h. The cells were collected and treated with pre-cooled RIPA lysis buffer for 15 min on ice. Ten percent of the lysate was used as the input, while the remainder was used for labeling serotonylated proteins. To detect GPX4 serotonylation, HEK293T cells were transfected with the plasmid, and the cells were harvested and lysed 48 h later, after which 5-PT (500 μM) was added to the lysate for incubation at 37 °C for 2 h. To determine whether GPX4 serotonylation in HGC-27 cells can be inhibited by SERT inhibitors, the cells were incubated with 5-PT (100 μM) combined with sertraline (Shyuanye, S71403) or fluoxetine (Aladdin, F131623) for 24 h. The cells were collected and treated with pre-cooled RIPA lysis buffer for 15 min on ice. Ten percent of the lysate was used as the input, while the remainder was used to detect GPX4 serotonylation.

5-PT was conjugated with an azide-biotin molecule via copper-catalyzed click chemistry. Specifically, azide-biotin (Vectorlabs, CCT-1265, 100 μM) was added to the sample, followed by the addition of TBTA (Sigma‒Aldrich, 678937, 100 μM), TCEP (Sigma‒Aldrich, C4706, 1 mM), CuSO_4_ (Sigma‒Aldrich, C1297, 1 mM), and sodium ascorbate (Sigma‒Aldrich, 11140, 2.5 mM). The mixture was allowed to react at room temperature for 2 h. After the labeling reaction was complete, the proteins were precipitated with pre-cooled methanol and stored at –80 °C overnight. Next, the samples were centrifuged at 8000 × *g* for 10 min at 4 °C, the supernatant was discarded, and the protein pellets were washed with pre-cooled methanol. The protein pellets were subsequently resuspended in 0.5% SDS buffer. Streptavidin agarose beads were added to the samples, which were rotated at room temperature for 2 h. The beads were collected, washed with 8 M urea to remove non-covalently bound proteins, and then washed three times with IP buffer^29^. The washed beads were suspended in 1× SDS loading buffer and heated to 99 °C for 10 min. SDS‒PAGE and western blotting were performed on both the IP and input samples to detect serotonylated proteins.

### MDC assays

To evaluate GPX4 serotonylation, GPX4 (10 μg) and TGM2 (0.25 μg) proteins were added to an enzymatic buffer (25 mM Tris-Cl (pH 8) and 5 mM CaCl_2_ plus protease inhibitors), followed by the addition of MDC (Sigma‒Aldrich, D4008, 5 mM) or 5-HT (5 mM). The serotonylation reaction was competed with the TGM2 inhibitor cystamine (Sigma‒Aldrich, C121509, 4 mM) or excess 5-HT (5 mM). The mixture was subsequently incubated at room temperature for 3 h in the dark and then heated to 99 °C for 10 min. Next, the samples were separated by SDS‒PAGE. Serotonylated proteins were visualized under UV light, followed by Coomassie Brilliant Blue (CBB) staining to assess protein loading^18,23^.

### Identification of GPX4 serotonylation sites

A buffer containing 50 mM HEPES, 10 mM CaCl₂, and protease inhibitor (1×) was prepared. For the 50 μL TGM2 incubation system, the following components were added: 5 μL of recombinant protein (1 μg/μL), 2 μL TGM2 (commercially sourced, diluted to 0.5 μg/μL), 5 μL of 100 mM 5-HT, and 38 μL of buffer. The mixture was incubated at 37 °C with shaking in the dark for 3 h, and the reaction was terminated by incubation at 70 °C for 10 min. Protein digestion was performed using the FASP method. Briefly, protein solutions were loaded into 10 kDa molecular weight cutoff ultrafiltration devices and centrifuged at 12,000× *g* for 10 min at 4 °C to remove the flow-through. The retained material was subsequently washed once with NH₄HCO₃ buffer via centrifugation. Afterward, 100 μL of NH₄HCO₃ and 0.5 μg of trypsin were added, followed by incubation at 37 °C for 16 h. After digestion, the samples were centrifuged at 12,000× *g* for 10 min at 4 °C, and the supernatant was collected. The centrifugation step was repeated after fresh NH₄HCO₃ was added. The supernatants from both centrifugation steps were combined to obtain the peptide solution, which was acidified with 1% formic acid (FA). Peptides were desalted using C18 StageTips, dried under vacuum, reconstituted in 0.1% FA, and quantified for subsequent LC‒MS analysis.

Peptides (800 ng per sample) were separated chromatographically using a Vanquish Neo UHPLC system (Thermo Scientific). The mobile phases were A) 0.1% formic acid in water and B) 0.1% formic acid in 80% acetonitrile. The column was equilibrated with 96% mobile phase A. Samples were loaded onto a trap column (PepMap Neo 5 μm C18, 300 μm × 5 mm; Thermo Scientific) and separated on an analytical column (μPAC Neo High Throughput column; Thermo Scientific) with the following gradient: 0–0.1 min, 4%–8% B; 0.1–0.7 min, 8%–12% B; 0.7–10.4 min, 12%–28% B; 10.4–14 min, 28%–45% B; 14–14.2 min, 45%–99% B; and 14.2–15 min, 99% B. Eluted peptides were analyzed with an Orbitrap Astral mass spectrometer (Thermo Scientific) in data-dependent acquisition (DDA) mode over a 15-min period. The parameters included an electrospray voltage of 2.2 kV, detection in positive ion mode, a precursor scan range of 350–1500 m/z, a full MS resolution of 240,000, an automatic gain control (AGC) target of 500%, and a maximum injection time of 3 ms. For MS/MS analysis, the resolution was 80,000, the AGC target was 500%, the maximum injection time was 3 ms, the RF lens was 40%, higher energy collisional dissociation (HCD) activation was applied, the isolation window was 2 Th, the normalized collision energy was 25%, and the cycle time was 0.6 s. Database searches were performed with pFind 3.2.0.

### Isotopic labeling GPX4 serotonylation assay

Each 50 μL reaction mixture contained 1× 50 mM HEPES reaction buffer (1× protease inhibitor), 5 mM 5-HT-*d*_4_ (MCE, HY-B1473S) or 5 mM 5-HT combined with 5 mM CaCl_2_, 1.25 μg TGM2, and GPX4 recombinant protein (50 μg) and was established on ice. The mixture was incubated at 37 °C with shaking in the dark for 3 h. Then, 20–30 mg of urea (final concentration of 6 M) was added to denature the proteins, TCEP (final concentration of 10 mM) was added for a 30 min incubation at 37 °C to reduce disulfides, and iodoacetamide (final concentration of 30 mM; final volume of 80 μL) was added for 30 min at room temperature in the dark to alkylate the reduced thiols. The solution was diluted to 2 M urea with 100 mM ammonium bicarbonate in H_2_O (final volume of 240 μL) and subjected to trypsin (sequence grade, Promega) digestion at a 1:25 trypsin:protein ratio (w:w) overnight (~16 h) at 37 °C. The resulting peptide solution was acidified with FA at a final concentration of 5%, desalted, dried with a SpeedVac and reconstituted for peptide fractionation.

### LC-MS/MS proteomics analysis (isotopic labeling)

Five hundred nanograms of digested sample from each treatment group were injected in technical triplicates. The peptides were analyzed using a nanoElute ultra-high-performance liquid chromatography (UHPLC) system (Bruker) coupled to a timsTOF Pro 2 mass spectrometer (Bruker) and were separated at a flow rate of 300 nL/min using a 60-min gradient on a 20-cm analytical column (75 μm ID, 1.9 μm, C18 beads, made in-house). Mobile phase B consisted of 0.1% formic acid in acetonitrile (ACN), and gradient elution was performed with three linear segments: from 2% to 22% over 40 min, from 22% to 37% over 10 min, and from 37% to 80% over 5 min, after which 80% mobile phase B was maintained for an additional 5 min to wash the analytical column. All separation processes were performed in an integrated heated column oven at 50 °C. DDA was performed in PASEF18 mode with 10 PASEF MS/MS scans. The capillary voltage was set to 1500 V, and the spectra were acquired in the range of m/z from 100 to 1700 Th with an ion mobility range (1/K0) of 0.6 to 1.6 Vs/cm^2^. The ramp and accumulation times were set to 100 ms to achieve a duty cycle close to 100% and a total cycle time of 1.1 s. The collision energy was increased linearly as a function of mobility from 59 eV at 1/K0 = 1.6 Vs/cm^2^ to 20 eV at 1/K0 = 0.6 Vs/cm^2^. Precursors with charge states from 0 to 5 (a peptide precursor charge state of 0 indicates that the isotope ion was not detected for that peptide precursor) were selected with a target value of 20,000 and an intensity threshold of 5000. Any precursors that reached the target value in arbitrary units were dynamically excluded for 0.4 min.

### Proteomic data analysis (isotopic labeling)

The raw MS data files were searched directly with Byonic v5.11.4 (Protein Metrics) against the human proteome fasta (UP000005640). Peptides were required to have tryptic C-terminus cleavage, and up to two missed cleavages were allowed. The precursor and product ion mass tolerances were set to 10 ppm and 50 ppm, respectively. Carbamidomethylation of cysteine residues (+57.02 Da) was set as a fixed modification. Oxidation of methionine residues (+15.99 Da) was included as a common variable modification (up to two per peptide). Serotonylation of glutamine was included as a rare variable modification: +159.06 Da (5-HT-light) or +163.09 Da (5-HT-heavy).

### RNA extraction and real-time qPCR

Total RNA was extracted according to the manufacturer’s instructions using the RNA Isolater Total RNA Extraction Reagent (Vazyme, R401-01-AA). The extracted RNA was reverse-transcribed into cDNA using the HiScript II 1st Strand cDNA Synthesis Kit (Vazyme, R223-01). Real-time qPCR was performed using the QuantStudio Real-time PCR system (Life Technologies) and SYBR Green qPCR Master Mix (Yeasen, 11203ES03). Relative mRNA expression was calculated using the 2^−ΔΔCt^ method and normalized to the mRNA level of GAPDH [18]. The following primers were used in this study:

Tgm1 forward, 5′-TCTGGGCTCGTTGTTGTGG-3′;

Tgm1 reverse, 5′-AACCAGCATTCCCTCTCGGAT-3′;

Tgm2 forward, 5′-GACAATGTGGAGGAGGGATCT-3′;

Tgm2 reverse, 5′-CTCTAGGCTGAGACGGTACAG-3′;

Tgm3-forward, 5′-CTGGCAGGTGCCTATGAATCG-3′;

Tgm3-reverse, 5′-TCCAGACTCAAGACTTCGGTT-3′;

Tgm4 forward, 5′-AGACCAAGAAGCTCGTGCTG-3′;

Tgm4 reverse, 5′-GAGGTTGGAGAGGTCGGTTC-3′;

Tgm5 forward, 5′-TGCCCCATCACAGGACTAGAG-3′;

Tgm5 reverse, 5′-ACAAACATGATACTGTCCATGCC-3′;

Tgm6 forward, 5′-ACACCCAAGATTACCCTTGCT-3′;

Tgm6 reverse, 5′-GGATGCGTGAGGTCCTGTC-3′;

Tgm7 forward, 5′-TCCCCAGGAACAACAAGGAC-3′;

Tgm7 reverse, 5′-CCAGACGTTCTCAGGTCGG-3′;

Gapdh forward, 5′-TGTGTCCGTCGTGGATCTGA-3′;

Gapdh reverse, 5′-CCTGCTTCACCACCTTCTTGA-3′;

GPX4 forward, 5′-GAGGCAAGACCGAAGTAAACTAC-3′;

GPX4 reverse, 5′-CCGAACTGGTTACACGGGAA-3′.

### Indirect immunofluorescence assay (IFA)

For the IFA, MFC cells were seeded on glass coverslips and cultured under standard conditions. Cells were incubated with the clickable serotonin analog 5-propargyltryptamine (5-PT; 100 μM) for the indicated durations (0.5 h, 1 h, 2 h, 4 h, 8 h, 12 h, or 24 h), with an untreated group included as a negative control. Samples were imaged by fluorescence microscopy to visualize the time-dependent intracellular accumulation of 5-PT (Azide-545 signal). To assess the contribution of different monoamine and serotonin transporters to serotonin uptake, cells were pretreated for 12 h with selective pharmacological inhibitors targeting individual transporters, including nisoxetine (MCE, HY-B1704A), GBR-12909 (MCE, HY-13217), sertraline (Shyuanye, S71403) or fluoxetine (Aladdin, F131623), tetrabenazine (Shyuanye, S80813), and decynium-22 (MCE, HY-107740). Cells were then incubated with the clickable serotonin analog 5-propargyltryptamine (5-PT) for 0.5 h, followed by click chemistry-based labeling and downstream immunofluorescence or biochemical analyses to evaluate intracellular serotonin uptake. The cells were washed with pre-cooled PBS and then fixed with 4% paraformaldehyde (PFA) solution at 4 °C for 15 min. Then, the cells were permeabilized with 0.1% Triton X-100 and blocked with 3% BSA at room temperature for 1 h. Subsequently, a mixture of Azide-545 (100 μM), TBTA (100 μM), TCEP (1 mM), CuSO_4_ (1 mM), and sodium ascorbate (2.5 mM) in PBS was prepared and incubated with the samples at 37 °C in the dark for 2 h. Next, the samples were washed three times with PBS, after which the nuclear DNA was stained with DAPI. Cell images were captured using a Zeiss LSM880 confocal microscope with a 63× oil immersion lens.

### Protein purification

Proteins were purified using the *E. coli* BL21 (DE3) strain (Beyotime, D1015S). BL21 (DE3) cells were transformed with plasmids encoding N-terminal 6×His-tagged GPX4 or TGM2. Protein expression was induced in BL21 cells (OD600 = 0.6) with 0.25 mM isopropyl-*β*-D-thiogalactopyranoside (IPTG) at 22 °C for 20 h. Collected bacteria were lysed in lysis buffer (20 mM Tris HCl, pH 7.5, 500 mM NaCl, 10% glycerol, and 1 mM DTT) with a high-pressure homogenizer, followed by centrifugation at 17,000× *g* for 30 min at 4 °C. The supernatants were incubated with Ni-NTA agarose beads (Yeasen, 20504ES08) for affinity purification according to the manufacturer’s instructions. Protein purity was determined by CBB staining, and protein concentrations were determined by a Bradford protein assay^52^.

### Protein stability assay

The GPX4 (WT) and GPX4 (mutant) expression plasmids were transfected into HEK293T cells for 48 h, followed by treatment with CHX (MCE, HY-12320) at the indicated time points. GPX4 levels were subsequently quantified at different time points.

### Plasma sample collection, metabolite extraction and untargeted metabolomics analysis

Plasma samples from GC patients and healthy controls were collected for untargeted metabolomic profiling conducted by Majorbio (Majorbio Biotech Co., Ltd., Shanghai, China). Peripheral blood was processed by sequential centrifugation at 1000× *g* for 10 min and 2000× *g* for 5 min at 4 °C to obtain plasma, which was stored at −80 °C until analysis. For metabolite extraction, 100 μL of plasma was combined with 400 μL of cold extraction solvent (acetonitrile/methanol, 1/1, v/v) supplemented with 0.02 mg/mL L-2-chlorophenylalanine as an internal standard, and the samples were briefly mixed and subjected to low-temperature ultrasonication at 5 °C for 30 min (40 kHz). The samples were then incubated at −20 °C for 30 min to promote protein precipitation, followed by centrifugation at 13,000× *g* for 15 min at 4 °C. The resulting supernatant was evaporated under a nitrogen stream, and the dried residue was reconstituted in 100 μL of acetonitrile:water (1:1, v/v), sonicated for 5 min at 5 °C, and centrifuged again at 13,000× *g* for 10 min at 4 °C. The clarified extracts were transferred to autosampler vials for LC-MS/MS analysis. A pooled quality control (QC) sample prepared by mixing equal aliquots of all plasma samples was analyzed in parallel and injected at regular intervals (every five samples) to assess analytical stability. Untargeted LC-MS/MS analysis was performed on a Thermo UHPLC-Q Exactive HF-X platform equipped with an ACQUITY HSS T3 column (100 × 2.1 mm, 1.8 μm; Waters, USA), with data acquired in DDA mode.

### Analysis of raw LC-MS/MS data

The raw LC-MS/MS data were processed using Progenesis QI (Waters Corporation, Milford, USA) software, generating a three-dimensional data matrix in CSV format that contained sample information, metabolite names, and mass spectral response intensities. Internal standard peaks, as well as false positives (e.g., noise, column bleed, and derivatized reagent peaks), were removed from the matrix, followed by data reduction and peak pooling. Metabolites were identified by searching the HMDB (http://www.hmdb.ca/), METLIN (https://metlin.scripps.edu/), and Majorbio databases. A volcano plot of the detected metabolites in GC patients versus NGC controls was constructed in this study. The data were analyzed by a two-sided Wilcoxon rank-sum test followed by the Benjamini–Hochberg (BH) multiple comparison test with a false discovery rate (FDR) < 0.05 and a FC > 1.2 or < 0.83.

### GC patient specimens

The patients included in the study provided written informed consent for the use of their specimens. The studies were performed in accordance with the Declaration of Helsinki and approved by the Huashan Hospital Institutional Review Board (HIRB), Fudan University (Approval No. 2017-222). Plasma samples were collected from 10 healthy individuals and 41 GC patients for untargeted metabolomics. Seven tumor tissues from GC patients were collected for western blot analysis. Tumor tissues and normal tissues were taken from patients with GC during surgery after providing informed consent.

### Bioinformatics analysis

The relative mRNA expression levels of GPX4 and TGM2 in the GSE54129 dataset from the GEO database (https://www.ncbi.nlm.nih.gov/geo/) were analyzed. Kaplan–Meier survival analysis of GC patients with low or high TGM2 expression was performed with KM plotter. Publicly available transcriptomic datasets from TCGA-STAD (human) and a mouse gastric cancer dataset (GSE199261) were used to assess the expression of additional components of the serotonin system.

### Statistical analysis

All experiments were performed 2–3 times. All data were expressed as mean ± SEM or SD and analyzed using Student’s *t* test or ordinary one-way ANOVA or 2-way ANOVA. *P* < 0.05 was considered statistically significant, whereas ‘ns’ indicates not significant. All statistical analyses were performed using GraphPad Prism v10.3.1.

## Supplementary information

Supplemental table 1-2

Supplementary Information

## Data Availability

The data that support the findings of this study are available from the corresponding author upon reasonable request.
