# Supplementary material for: TGM2-mediated serotonylation of GPX4 confers ferroptosis resistance to promote gastric tumorigenesis
Source: Cell Discov. 2026 Apr 28;12:30. doi: 10.1038/s41421-026-00885-6 (PMC13125317; doi:10.1038/s41421-026-00885-6)
Supplement: Supplementary file 2 — Supplementary Information [file 41421_2026_885_MOESM2_ESM.pdf]

TGM2-Mediated Serotonylation of GPX4 Confers Ferroptosis Resistance to Promote Gastric Tumorigenesis

Junping Bai, Dandan Geng, Xinwen Chen, Wanting Li, Xiaowei Wang, Luyang Tian, Yi Han, Zhao Jin, Meihang Du, Yang Tang, Weisheng Hu, Chunxiao Zhu, Shan Zhang, Zhangting Zhao, Run Zhang, Xinru Zhang, Wei Kang, KaFai To, Sachiyo Nomura, Fenghua Guo, Shi Jiao, Yixuan Xie, Zhaocai Zhou, Chao Dong, Hui Li, Liwei An

Supplementary figures and legends

Supplementary Fig S1. 5-HT promotes gastric cancer cell growth via TGM2.

Supplementary Fig S2. Chemoproteomic profiling of protein serotonylation in GC.

Supplementary Fig S3. Validation of ferroptosis-associated candidates with serotonylation.

Supplementary Fig S4. GPX4 is specifically serotonylated at Gln55 and Gln77 residues.

Fig 5. GPX4 serotonylation stabilizes its protein stability to confer ferroptosis resistance.

Supplementary Tables

Supplementary Table S1. A total of 1,135 metabolites identified in healthy donor and GC patients.

Supplementary Tables S2. A list of 861 seronolytated proteins identified in MFC gastric cancer cell.

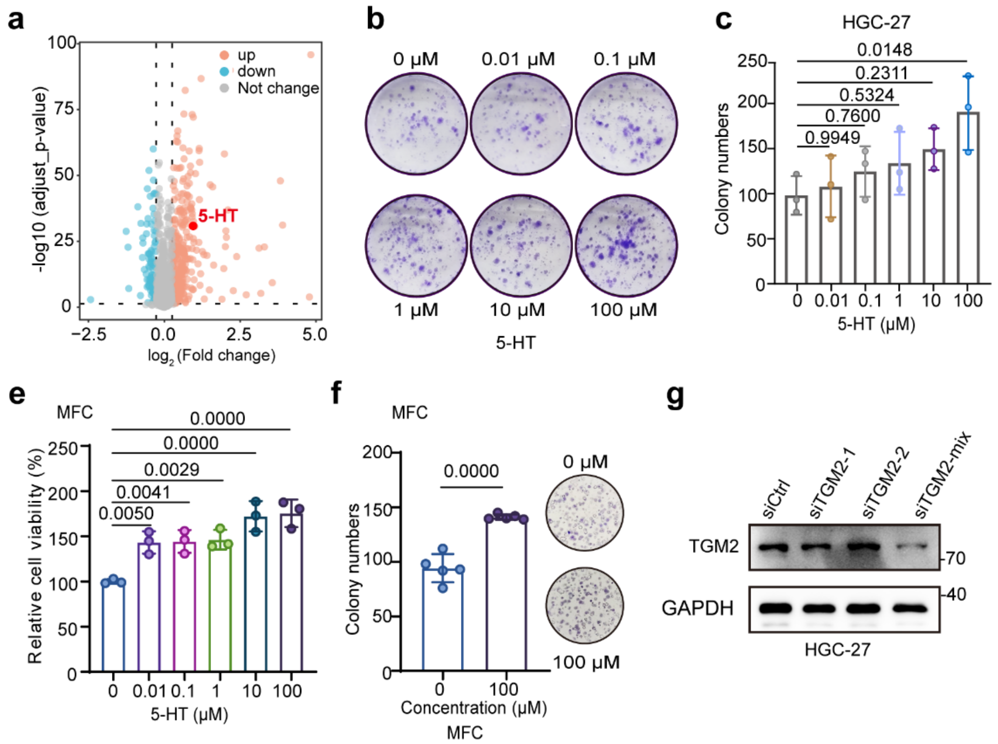

Supplementary Fig S1. 5-HT promotes gastric cancer cell growth via TGM2. a Volcano plot of the detected metabolites in plasma metabolomics (GC patients versus NGC controls) in this study. Two-sided Wilcoxon rank-sum test followed by Benjamini–Hochberg (BH) multiple comparison test with false discovery rate (FDR) < 0.05 and fold change (FC) > 1.2 or < 0.83. **b** Representative images of colony formation assay performed after treatment with various concentrations of 5-HT for 8 days. **c** Colony formation assay of HGC-27 cells was performed after treatment with various concentrations of 5-HT for 8 days (n = 3 per group). **d** 5-HT promotes the proliferation of AGS cells *in vitro*. After 6-hour serum starvation, AGS cells were treated with various concentrations of 5-HT for 48 hours to assess their relative cell viability (n = 3 per group). **e** 5-HT promotes the proliferation of MFC cells *in vitro*. After 6-hour serum starvation, MFC cells were treated with various concentrations of 5-HT for 48 hours to assess their relative cell viability (n = 3 per group). **f** Colony formation assay of MFC cells was performed after treatment with 5-HT for 8 days (n = 5 per group). **g** Western blot analyses confirming the protein expression levels of TGM2 in HGC-27 cells transfected with indicated siRNAs. Data are presented as mean ± SD from two or three independent experiments. Statistical analysis was determined using Ordinary one-way ANOVA (c, d, e) and unpaired *t* test (f).

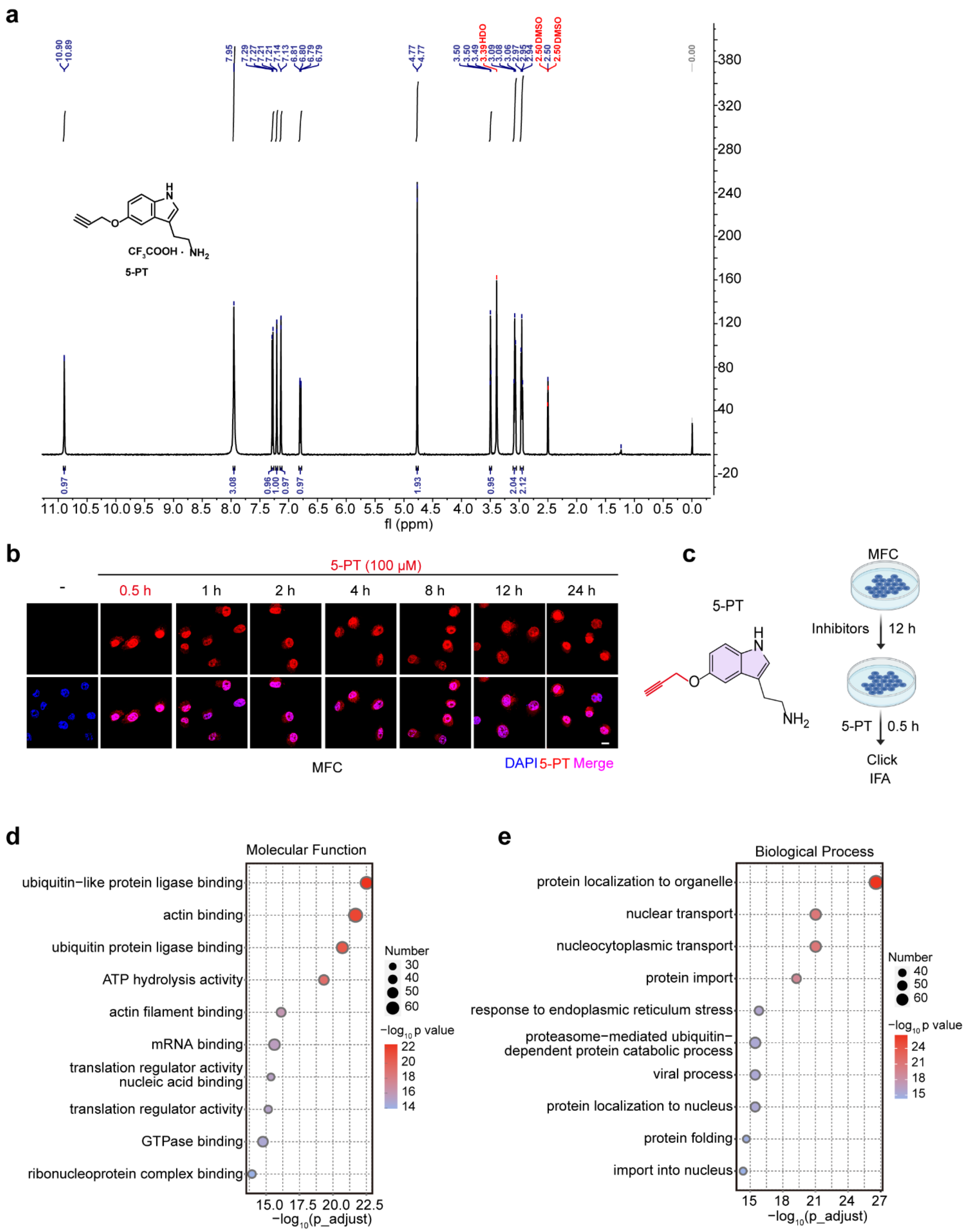

Supplementary Fig S2. Chemoproteomic profiling of protein serotonylation in GC. a Copies of ^{1}H-NMR Spectra (5-PT). **b** Cells were incubated with 5-PT for the indicated times (0.5–24 h), followed by click-based immunofluorescence labeling. Red, 5-PT signal; blue, DAPI nuclear staining. Scale bar, 10

μm. **c** Schematic illustration of the experimental design for transporter inhibition assays. Cells were pretreated with specific transporter inhibitors for 12 h, followed by incubation with 5-PT for 0.5 h prior to click-based immunofluorescence analysis. **d, e** Bioinformatics analysis of the 861 serotonylation proteins identified in GC. Gene Ontology (GO) molecular function (c) and Gene Ontology (GO) biological process (d).

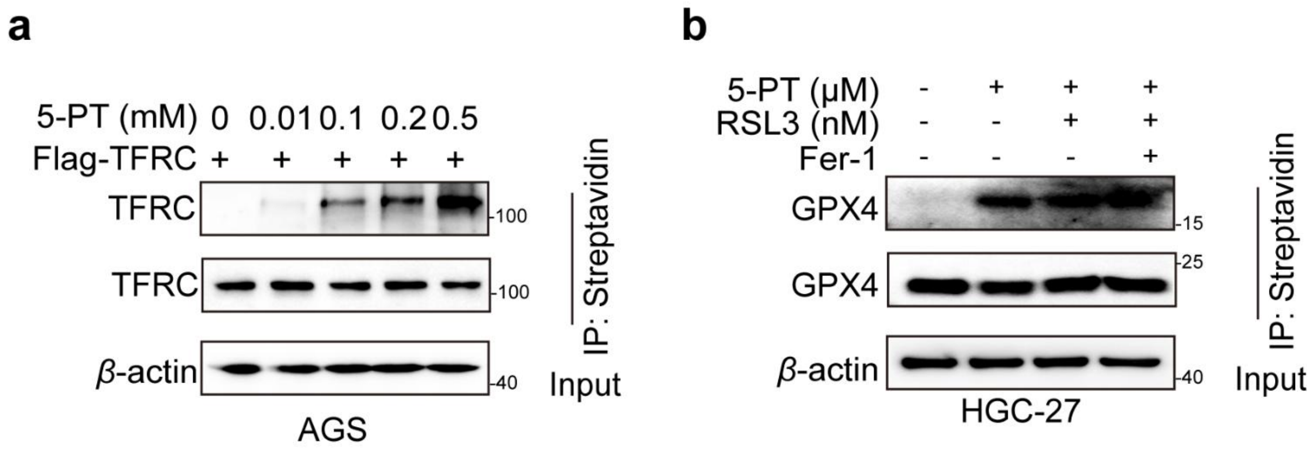

Supplementary Fig S3. Validation of ferroptosis-associated candidates with serotonylation. a 5-PT-based click chemistry for detection of serotonylation of ferroptosis-associated candidate proteins in AGS cells. **b** 5-PT-based click chemistry for detection of GPX4 serotonylation status in response to ferroptosis induction, with or without cell-death inhibitors.

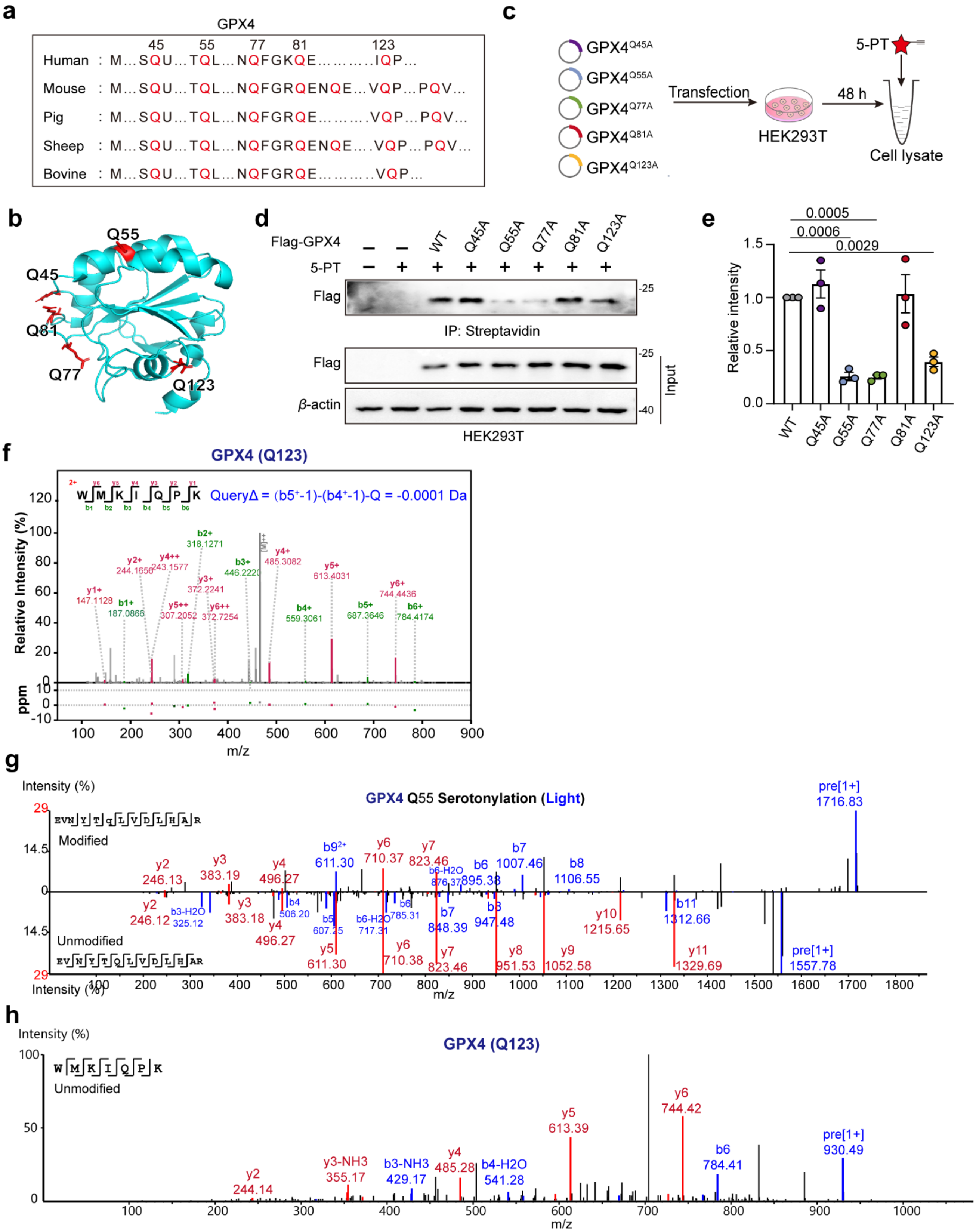

Supplementary Fig S4. GPX4 is specifically serotonylated at Gln55 and Gln77 residues. a Analysis of glutamine (Q) residues of GPX4 sequence across different species. Gln45, Gln55, Gln77, Gln81, and Gln123 were conserved in different species. **b** Localization of glutamine (Q) residues within the

human GPX4 crystal structure (PDB: 2OBI). **c** Workflow for assessing the serotonylated residues of GPX4 in HEK293T. **d** 5-PT-based click chemistry for detection of GPX4 serotonylation between WT- and point mutations in HEK293T cells. **e** Relative quantification of serotonylation intensity analysis of GPX4 (WT) and its mutants in HEK293T cells (mean ± SEM, Ordinary one-way ANOVA, n = 3). **f** MS/MS spectrum of the GPX4 peptide containing Q123, showing no detectable serotonylation, serving as a negative control. **g** MS/MS spectrum showing the fragmentation pattern of GPX4 at the serotonylated site-Q55, confirming the presence of 5-HT modification (Light) in SILAC experimental. **h** MS/MS spectrum of the GPX4 peptide containing Q123, showing no detectable serotonylation, serving as a negative control in SILAC experiment.

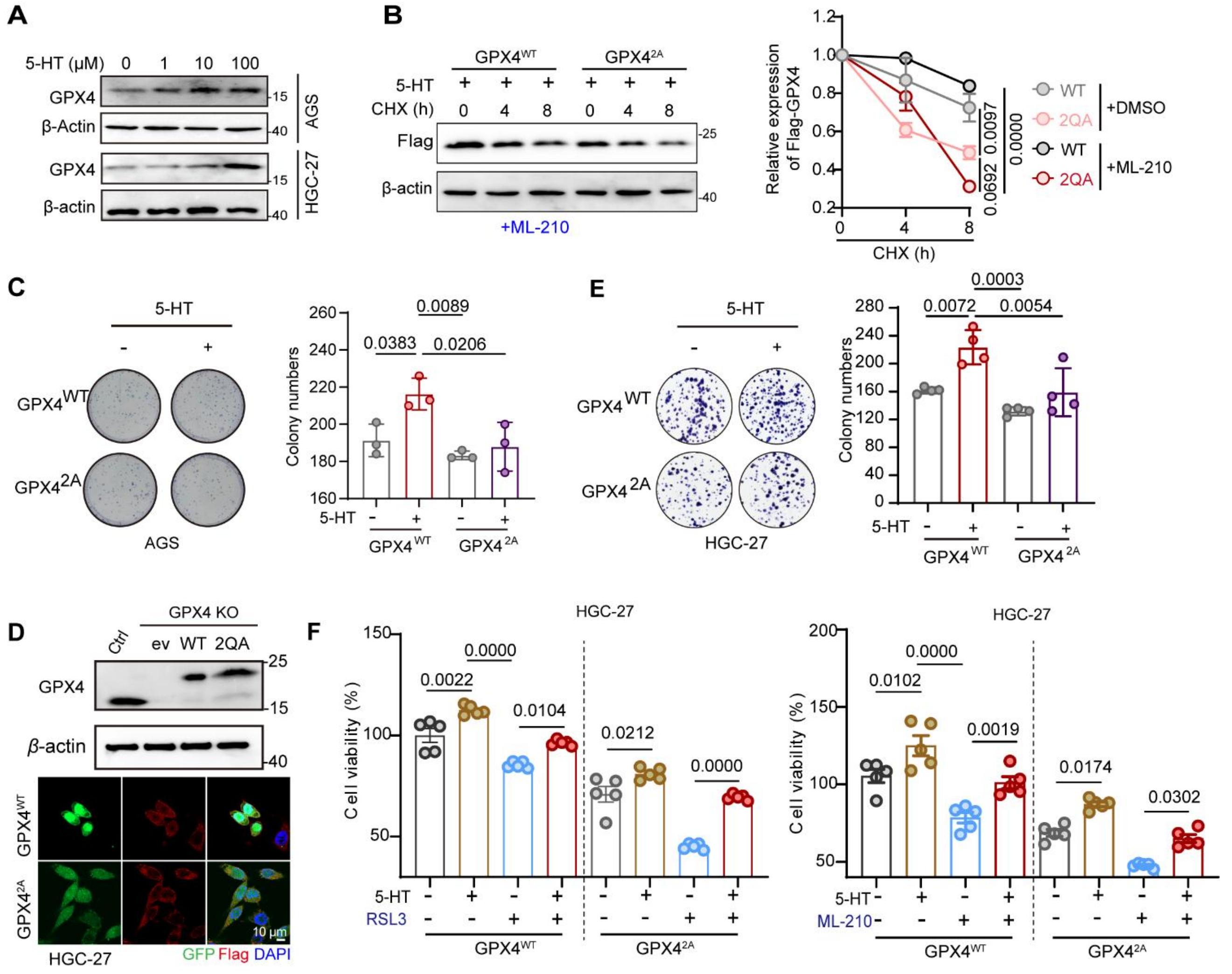

Supplementary Fig S5. GPX4 serotonylation stabilizes its protein stability to confer ferroptosis resistance. a Western blotting analysis of GPX4 protein level after treatment with different concentrations of 5-HT for 24 hours in HGC-27 and AGS cells. **b** HEK293T cells transfected with $GPX4^{WT}$ or $GPX4^{2A}$ were treated with 5-HT (100 μM) and analyzed using CHX (50 μM) chase experiments at different time points. Cycloheximide (CHX) chase assay analyzing the protein stability of $GPX4^{WT}$ and $GPX4^{2A}$ in the presence of ferroptosis inducers (ML-210). The quantification of GPX4 protein levels (Flag) is shown on the right. **c** Colony formation assay was performed to assess the proliferation of AGS cells stably expressing $GPX4^{WT}$ or $GPX4^{2A}$, treated with or without 5-HT (10 μM) (n = 3). Left: representative images; Right: quantitative analysis. **d** Validation of exogenous GPX4 transfection efficiency in GPX4 KO HGC-27 cells. Immunofluorescence images (on the bottom) of HGC-27 cells stably expressing $GPX4^{WT}$ or $GPX4^{2A}$. Cells were stained with DAPI (blue), GFP (green), and Flag-GPX4 (red). Scale bars, 10 μm. **e** Colony formation assay was performed to assess the proliferation of AGS cells stably expressing $GPX4^{WT}$ or $GPX4^{2A}$, treated with or without 5-HT (10 μM) (n = 3). Left:

representative images; Right: quantitative analysis. **f** Cell viability of HGC-27 cells transfected with $GPX4^{WT}$ or $GPX4^{2A}$ was measured following treatment with 5-HT (10 μM) and RSL3 (500 nM) or ML-210 (5 μM) for 24 hours (n = 5). Data are presented as mean ± SD from two or three independent experiments. Statistical analysis was determined using by Ordinary one-way ANOVA (c, e).
